# Listening to Cellular Whispers through Glycosphingolipids: A Comprehensive Review of Recent Advancements in Glycosphingolipid Analysis and Annotation

**DOI:** 10.1021/acs.analchem.5c08130

**Published:** 2026-04-17

**Authors:** Amirreza Dowlati Beirami, Evelyn Rampler

**Affiliations:** † Institute of Analytical Chemistry, Faculty of Chemistry, University of Vienna, Waehringer Str. 38, 1090 Vienna, Austria; ‡ Vienna Doctoral School in Chemistry (DoSChem), University of Vienna, Waehringer Str. 42, 1090 Vienna, Austria

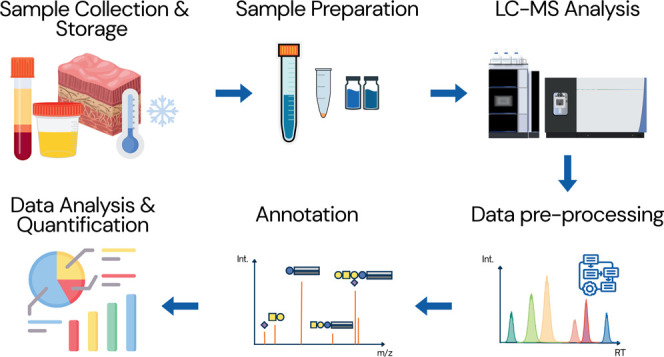

## 1 Introduction

According to the LIPID MAPS Structure Database, there are eight main categories of lipids: fatty acyls, glycerolipids, glycerophospholipids, sphingolipids, prenol lipids, sterol lipids, saccharolipids, and polyketides, together comprising over 48,000 unique structures.^1–3^ However, this is likely only a fraction of the potential lipid diversity in nature. Within this diversity, glycosphingolipids (GSLs), in the sphingolipid class, stand out as one of the most structurally diverse molecules due to variations in both their glycan headgroups and their ceramide backbones. The glycan component can include a range of sugars like glucose and galactose and in different arrangements and branching patterns^4^ and often with additional modifications. The ceramide tail also varies widely, with differences in fatty acid chain length, saturation, methylation, and hydroxylation.^5–8^ Much like LEGO bricks, these structural subunits can be used to build an enormous number of possible GSLs, with the diversity of oligosaccharides alone exceeding Avogadro’s number.^4^ This complexity (shown in Figure 1) makes it challenging to accurately characterize GSLs, especially around their average molecular mass, highlighting the need for detailed analysis to understand their role in cell structure and function.

**Figure 1:**
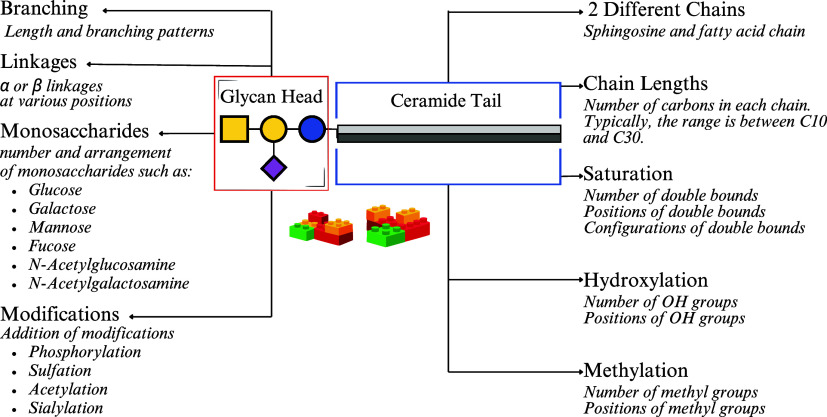
Overview of glycosphingolipid diversity, showing the combinatorial complexity of glycan heads and ceramide tails that create a wide variety of structures.

GSLs are most extensively studied in humans and vertebrates, but their presence is not limited to higher eukaryotes. GSLs and structurally related analogs have been identified in a wide range of organisms, including fungi,^9^ plants,^10^ bacteria,^11^ and even viruses that infect planktonic hosts.^12^ Although there is extensive knowledge of the distribution of GSLs, the majority of our understanding regarding their biosynthesis, structural variety, and biological roles comes from studies conducted on eukaryotic systems. In these systems, GSLs play crucial roles in signaling pathways, membrane structure, and immune response regulation. Vertebrates among eukaryotes exhibit the greatest variety and functional specialization of GSLs, especially within the central nervous system. These molecules play a crucial role in maintaining membrane stability and facilitating signal transmission and are linked to neurological functions such as memory, neuroplasticity, and cognitive processes.^13^ GSL species in vertebrates exhibit specific structural modifications adapted to defined physiological roles.^13,14^ Accordingly, this review focuses on eukaryotic GSLs while recognizing the need for expanded investigation into their noneukaryotic counterparts.

The diversification of GSLs at the biological level is crucial to their various functions, and any disruption of their regulation can significantly affect cellular operations. To obtain accurate insights into the structures and functions of GSLs, advanced analytical techniques are essential. Mass spectrometry (MS), and particularly high-resolution mass spectrometry (HRMS), has been a key player in enhancing our understanding of the molecular structures of GSLs and their roles in cellular activities.^15^ In combination with separation techniques such as high-performance liquid chromatography (HPLC), MS provides both structural elucidation and reliable quantification of intact GSLs, making LC–MS one of the most suitable methods for analyzing GSLs, as a key focus in this review. Previous reviews in GSL analysis have extensively covered MS-based ionization, fragmentation, and labeling strategies.^15–17^ This review goes beyond methodological description and into crucial considerations within the analytical cycle, integrating recent advances and approaches of GSL analysis with biological logic, analytical workflow reliability, and a strong focus on annotation strategies. Importantly, it introduces a critical point of view on GSL misannotation, highlighting common sources of structural ambiguity and overinterpretation of MS data, and even though significant challenges remain in fully characterizing GSL diversity, this review aims to address the best practices achieved so far.

### 1.1 GSL Biosynthesis and Classification

The classification of GSLs is primarily based on the glycan head. Based on this, GSLs are categorized into classes such as ganglio-, globo-, lacto-, muco-, and arthro-series.^18^ Gangliosides (ganglio-series), such as GM1, are conventionally designated using the Svennerholm nomenclature, which remains the most widely utilized abbreviated system for these GSLs. In this system, the prefix “G” denotes ganglioside, while the subsequent letter (M, D, T, or Q) indicates the number of sialic acid residues present in the glycan headgroup, corresponding to mono-, di-, tri-, or quadro-sialylated species, respectively. The numerical index reflects the relative length of the neutral oligosaccharide chain within the core structure of the ganglio-series (Galβ1–3GalNAcβ1–4Galβ1–4Glc-Cer); accordingly, the index increases from 1 as terminal monosaccharide units are absent, which can be conceptually expressed as (5 – *n*), where *n* signifies the number of sugars present in the glycan chain. Lowercase suffixes (e.g., a, b, c) further elucidate the positional distribution of sialic acid residues within the glycan chain, particularly their attachment to the inner galactose residue. For instance, GD1a contains two sialic acids, one linked to the internal galactose, while in GD1b, both sialic acids are connected to the inner galactose residue.^19,20^ While this nomenclature provided an efficient and practical framework for the early classification of gangliosides, it was originally intended as a simplified naming convention and therefore does not fully accommodate the expanding structural diversity of gangliosides revealed by modern analytical techniques.

The GSL biosynthesis pathway begins with the enzyme serine palmitoyltransferase (SPT), which catalyzes the condensation of serine and palmitoyl-CoA to form 3-ketodihydrosphingosine, marking the initial step in sphingolipid synthesis.^6^ This intermediate is reduced to sphinganine by 3-ketosphinganine reductase.^21^ Afterwards, ceramide synthases (CerS) acylate sphinganine’s primary amine with fatty acids to form ceramide.^22^ Ceramides in mammals are synthesized by six types of ceramide synthases (CERS1–6), each showing a preference for different fatty acyl chain lengths. This results in diverse ceramide structures, which influence membrane properties and tissue functions.^22,23^

Glycosylation of ceramide begins by either glucosylceramide synthase (GCS) or galactosylceramide synthase to form glucosylceramide (GlcCer) or galactosylceramide (GalCer), respectively.^24^ At the simplest level, GlcCer and GalCer serve as the precursors for complex GSLs synthesized in the Golgi apparatus.^6^ GlcCer is further extended to lactosylceramide (LacCer) by β-1,4-galactosyltransferase 5/6,^25^ which serves as the biological pathway branching point for synthesizing complex ganglio-series, globo-series, and lacto-series GSLs.^18^

The ganglio-series begins with the addition of sialic acid to LacCer by ST3 β-galactoside α-2,3-sialyltransferase 5,^26^ producing GM3, the simplest ganglioside. Notably, ST3 β-galactoside α-2,3-sialyltransferase 5 is also involved in the synthesis of GM4 by the sialylation of GalCer.^27^ GM3 is further elaborated to GM2 and GM1 through the stepwise action of β1,3-*N*-acetylgalactosaminyltransferase 1 (GM2/GD2 synthase)^28^ and β-1,3-galactosyltransferase (GM1 synthase),^29^ respectively, or GD3 and GT3 through the stepwise action of ST8 α-*N*-acetyl-neuraminide α-2,8-sialyltransferase 1 ^30^ and ST8 α-*N*-acetyl-neuraminide α-2,8-sialyltransferase 5,^31^ respectively. Figure 2 shows a simplified overview of GSLs (mostly ganglio-series) and key players in their metabolism.

**Figure 2:**
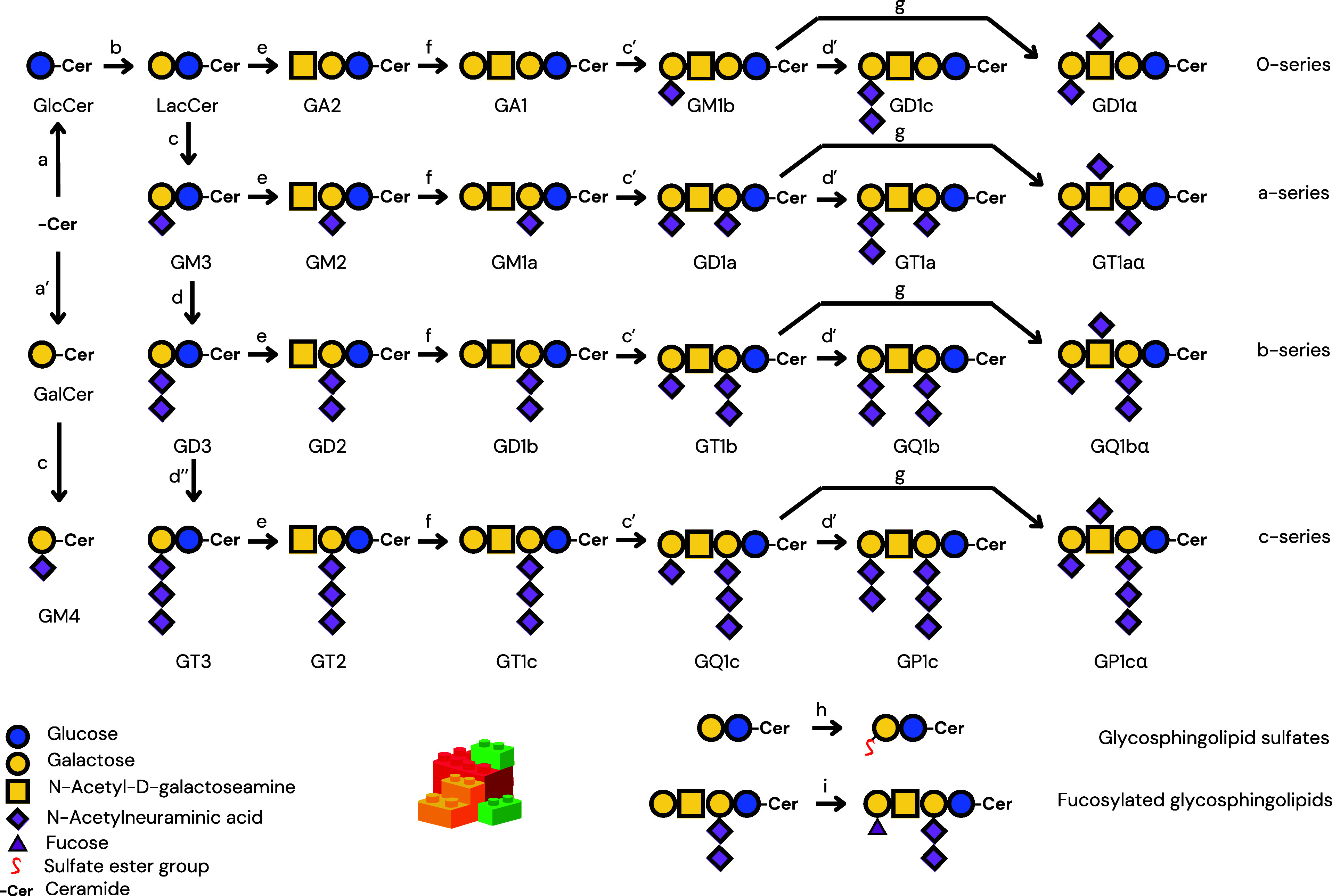
A simplified scheme of glycosphingolipid biosynthesis. The pathway highlights the LEGO-like assembly of GSLs through the sequential addition of monosaccharides and subsequent modifications. Further structural diversity arises from modifications, such as fucosylation and sulfation, in various GSL species. The enzymes involved in each biosynthetic step, to be identified from the literature, include the following: (a: UDP-glucose ceramide glucosyltransferase), (a′: UDP glycosyltransferase 8), (b: β-1,4-Galactosyltransferase 5/6), (c: ST3 β-galactoside α-2,3-sialyltransferase 5), (c′: ST3 β-galactoside α-2,3-sialyltransferase 2/3), (d: ST8 α-*N*-acetyl-neuraminide α-2,8-sialyltransferase 1), (d′: ST8 α-*N*-acetyl-neuraminide α-2,8-sialyltransferase 5), (d″: ST8 α-*N*-acetyl-neuraminide α-2,8-sialyltransferase 3/5), (e: β1,3-*N*-acetylgalactosaminyltransferase 1), (f: beta-1,3-galactosyltransferase), (g: ST6 *N*-acetylgalactosaminide alpha-2,6-sialyltransferase 4/5), (h: Galactose-3-*O*-sulfotransferase 1), and (i: fucosyltransferase family).^31–33^

The globo-series originates with the addition of galactose to LacCer by Gb3 synthase,^34^ followed by the incorporation of acetylgalactosamine (GalNAc) via Gb4 synthase and further GalNAc by Forssman synthase, generating the Forssman antigen.^35,36^ In parallel, if GalNAc is added to LacCer by Lc3 synthase, the lacto-series branch is formed,^37^ generating structures involved in cell adhesion and hormone clearance.

Additional structural variation in GSLs results from various modifications. One example is the action of fucosyltransferases, which add fucose residues that play a crucial role in forming Lewis blood group antigens and in regulating leukocyte adhesion.^38^ Likewise, sulfotransferases, such as galactose-3-*O*-sulfotransferase 1, catalyze the sulfation of galactosylceramide to generate sulfatides, which are essential for nervous system integrity. Identification of isoforms of these enzymes has resulted in the discovery of several therapeutic agents and targets.^39–42^ For instance, sulfatides (3′-sulfated galactosylceramides) are one of the major GSLs of myelin and are involved in nervous-system membrane organization and transmembrane signaling. They contribute to oligodendrocyte function, myelin stability, and axo–glial junction interactions.^43,44^ Altered sulfatide homeostasis has been linked to neurodegenerative and demyelinating conditions (including metachromatic leukodystrophy and multiple sclerosis), as well as broader disease contexts such as cancer and autoimmune disorders.^45^

The analysis of GSLs is challenging due to their structural complexity and low abundance in biological samples. The biosynthesis of GSLs resembles the assembly of molecular LEGO sets incorporating various glycan headgroups linked to a ceramide backbone. This modular structure creates a wide variety of possible configurations that can complicate analytical resolution. As a result, highly sensitive and selective methods are necessary to accurately resolve, identify, and quantify diverse and low-abundance GSL species within complex biological matrices.

Despite many technological advancements, there are still many challenges in comprehensive GSL profiling. Glycolipidomics faces challenges in analyzing GSLs due to a lack of standards and their limited coverage of lipid species. This limitation complicates both qualitative and quantitative analyses of GSLs, hampering a complete understanding of their biological functions.^46^ Furthermore, conventional techniques often struggle to differentiate between the structural isomers and isobars of GSL species. This distinction is essential for identifying the unique biological profiles linked to various diseases. To overcome these challenges, it is necessary to adopt innovative analytical strategies. These include enhanced extraction techniques, enrichment methods, improved chromatographic separations, advanced MS approaches, and comprehensive annotation approaches, all aimed at effectively addressing these limitations.^47,48^

### 1.2 Clinical Relevance of GSLs

As the field of glycobiology expands, the demand for diverse and structurally defined GSLs has grown, highlighting the clinical relevance of GSL analysis. For example, abnormal accumulation of GSLs in lysosomes can lead to storage disorders, known as GSL storage diseases, where the build-up disrupts normal cellular functions and affects various organs and tissues.^49^ Also, in cancer cells, highly abundant GSLs contribute to supporting tumor cell survival and progression, which underscores their potential as targets in cancer treatment strategies.^50^ Understanding the role of GSLs in health and disease has led to a promising treatment strategy of inhibiting GCS, an enzyme that is central to GSL biosynthesis. Inhibitors like venglustat reduce glucosylceramide levels, thus limiting GSL formation and ultimately providing potential benefits for disorders linked to abnormal GSL metabolism.^51^

The efficacy of GCS inhibition extends beyond disorders related to GSL metabolism. In this case, AL01211, a potent GCS inhibitor, has shown significant reductions in plasma glucosylceramide levels in preclinical studies.^49^ Clinical trials of venglustat have also confirmed its favorable safety and tolerability profiles, showing particular promise for managing GSL storage diseases, including Fabry and Gaucher diseases.^51,52^ Notably, AL01211 has similarly demonstrated an acceptable safety profile in early-phase studies.^49^

In addition to metabolic storage disorders, GCS inhibitors are also being studied for their potential in cancer therapy. Lower GSL synthesis has been shown to enhance antitumor immune responses. For example, UDP-glucose ceramide glucosyltransferase inhibitors like eliglustat increase the exposure of MHC and tumor antigen peptides to CD8^+^ T cells, potentially boosting immunotherapy outcomes.^50^ On the other hand, P-glycoprotein (P-gp) indirectly affects pathways critical for apoptosis and immune recognition by modulating GSL biosynthesis through the efflux of ceramide precursors, limiting their conversion into complex GSLs.^53^ These findings collectively support the importance of controlled GCS inhibition as a versatile and complex therapeutic strategy in both metabolic and oncologic contexts.

Given the integral roles of GSLs in health and disease, a comprehensive understanding of their synthesis, accumulation, and therapeutic targeting is essential. This review explores the main steps in intact GSL profiling workflows, including sample collection, preparation techniques, and advanced analytical methods, with a major focus on mass spectrometry and liquid chromatography (LC). The goal is to offer insights into the advancing analytical techniques and the broader implications of GSL analysis in both clinical and research environments.^54,55^

## 2 The Analytical Cycle: From Sample Preparation to GSL Detection

### 2.1 Sample Types in GSL Analysis

In GSL research, a range of samples, including serum, tissue, or specific cell culture samples, is used to track disease advancement or the effectiveness of therapeutic treatments and GSL changes during different conditions.^56–58^ Sample sources vary significantly based on research objectives and target analytes.

Human serum, plasma, and whole blood, readily accessible and stable matrixes, are frequently selected to analyze gangliosides and neutral GSLs. Dried blood spot samples have also been used in some studies due to good biomarker stability and cost-effectiveness.^59^ Human serum and plasma are important for studying biomarkers and detecting low-abundant sphingolipids, particularly in cancer research,^56,60^ cardiovascular^61,62^ and metabolic disorders like type 2 diabetes and morbid obesity,^63^ and genetic and neurological disorders,^64,65^ because they offer better comparability as a biological matrix.^66^

Specialized cell cultures, such as neuroblastoma (e.g., COG-N-683), pancreatic cancer (e.g., PSN1), and breast cancer cell lines (e.g., MDA-MB-231BR), provide an excellent platform for studying disease-specific GSLs like GM3, GD2, and GM1.^67^ Moreover, these controlled environments allow researchers to explore cancer-specific glycosylation mechanisms, supporting studies on cancer progression, metastasis, and response to treatment.^68^ In studies focused on metabolic syndromes, cell cultures also enable targeted investigations into lipid pathways impacted by metabolic shifts, as seen in liver cell cultures.^69^

Specific biological fluids, including amniotic fluid, placenta, milk, and maternal plasma, are essential in developmental and prenatal research, where they provide insights into lipids involved in fetal and maternal health.^70–74^ Lo et al. have shown that the GSL profile of extracellular vesicles secreted from human embryonic stem cells can help identify globo-series GSLs for monitoring embryo–maternal interactions, which may facilitate a healthy pregnancy.^73^

Tissue samples, although less accessible, provide crucial information about GSL distribution within organs. For instance, brain tissue is essential in neurodegenerative disease research, where the accumulation of specific gangliosides can be indicative of conditions like GM1 and GM2 gangliosidosis for Tay-Sachs and Sandhoff disease.^64,75^ Researchers also use liver^76^ and kidney tissues^77^ to study lipid storage, enabling direct analysis of lipid accumulation within affected organs. These studies often involve MS imaging, which maps lipid distribution at the cellular level, revealing pathological patterns unique to each tissue type.^75,78^ Lately, skin sampling by tape stripping has also been introduced for the investigation of lipid profiles of the stratum corneum.^79^

Regarding other biological fluids, cerebrospinal fluid (CSF) is sometimes used in neurological studies for its proximity to the central nervous system, and it offers a more direct reflection of brain lipid metabolism.^80^ However, CSF collection provides limited sample volumes, remains an invasive procedure, and is therefore less suitable for routine or repeated sampling, despite recent technical advances. On the other hand, urine samples can be collected easily in a noninvasive fashion and are also gaining recognition in GSL research for their potential to reveal biomarkers associated with conditions such as renal cell carcinoma and Fabry disease.^60,81^

Regarding the stability of GSLs, the study by Kim et al. stated that at −20 °C, most classes are stable in serum samples for up to 14 days; at 4 °C, all classes except GQ1 and Gb3 are stable for up to 7 days; and at 18 to 25 °C, all lipid classes except GD1 and GT2 were stable for up to 3 days, but in this condition, all GSL classes except LacCer became unstable by day 5.^82^ It can be assumed that dried GSL standards or samples have longer stability.

Plasma, cell cultures, tissue samples, and other biological fluids offer distinct advantages for GSL studies. The selection of the appropriate matrix is determined by logic involving the specific GSLs of interest and the research questions being explored. This variety gives researchers the freedom to explore GSL functions in health and disease in many different settings.

### 2.2 Sample Preparation Methods

In GSL research, effective sample preparation is the next crucial step for reliable analysis. Various methods and solvents were/are/need to be optimized based on the target analytes. Liquid–liquid extraction (LLE) with a chloroform/methanol/water mixture, known as Folch’s method^83^ for lipid extraction, is a commonly used approach for ganglioside and sulfatide analysis, as it effectively solubilizes these lipid molecules.^72,84–87^ This approach is known as the “gold standard” for the extraction of lipids^88^ and, compared to single-phase methanol extraction, can result in better identification, recovery, and peak intensity of sphingolipids.^67^ Another approach, which is well-known as the rapid lipid extraction method, is the Bligh and Dyer method.^89^ This method also uses chloroform, methanol, and water and has been used in some studies on GSL with slight modifications.^60^

A Soxhlet extraction method, which also uses a chloroform–methanol solvent system, can be an option in case there is a limited amount of starting material employed, because conventional extraction methods, such as the Folch method or the Bligh and Dyer method, might not be very successful in the determination and identification of very low amounts of GSL content.^90^

On the other hand, the methyl *tert*-butyl ether (MTBE) method has been investigated for its efficacy in extracting biosurfactant complexes, particularly glycolipids. MTBE-based extraction shows excellent enrichment for GSLs and is particularly known for its reduced toxicity compared to traditional chloro-organic solvents, making it suitable for large-scale studies.^91^ The process starts by mixing methanol and, afterward, MTBE with the sample to form a single-phase extraction solution. Next, water is introduced, causing the mixture to separate into two layers. The MTBE layer, enriched with lipids, stands in the upper phase, while the polar compounds, cell remains, and proteins settle in the lower layer. This separation minimizes contamination and eliminates the need for extra filtering steps.^92^

In addition, several methods, such as derivatization reactions and dissociation techniques, significantly enhance the structural resolution of GSLs, particularly for distinguishing isomeric species. Additional strategies, including permethylation, 2-(2-pyridilamino)-ethylamine (PAEA) labeling, and isobaric tagging, further expand analytical capabilities.^17,93,94^ In particular, Liyanage et al. showed that permethylation enhances the ionization efficiency of neutral GSLs, especially as [M + Na]^+^ ions in positive-ion mode, and facilitates MS2 acquisition of diagnostic ceramide-derived fragment ions, thereby improving the structural characterization of intact neutral GSLs.^93^ In another study, Barrientos and Zhang applied differential isotope labeling by permethylation using ^12^CH_3_I and ^13^CH_3_I combined with RPLC–MS/MS, enabling the relative quantification of intact glycolipids while simultaneously improving signal quality. Notably, permethylation was shown to substantially reduce analytical background in total lipid extracts by suppressing signals from highly abundant ester-linked lipids.^95^ Earlier work also demonstrated efficient methylation of GSLs using methyl sulfoxide, methyl iodide, and powdered NaOH, providing rapid and high-yield derivatization suitable for mass-spectrometric analysis of both neutral and acidic GSL species.^96^ As comprehensively reviewed by Hořejší and Holčapek,^15^ derivatization methods are also used to deepen the knowledge on structural isomers and better understand their biological relevance.

Each method and solvent choice reflects the specific analytical goals. The selection of the method must ensure that the target GSL profile is accurately captured. Figure 3 shows an overview of samples and sample preparation methods in recent studies, highlighting the variety of techniques and solvents that support GSL analyses across diverse biological samples.

**Figure 3:**
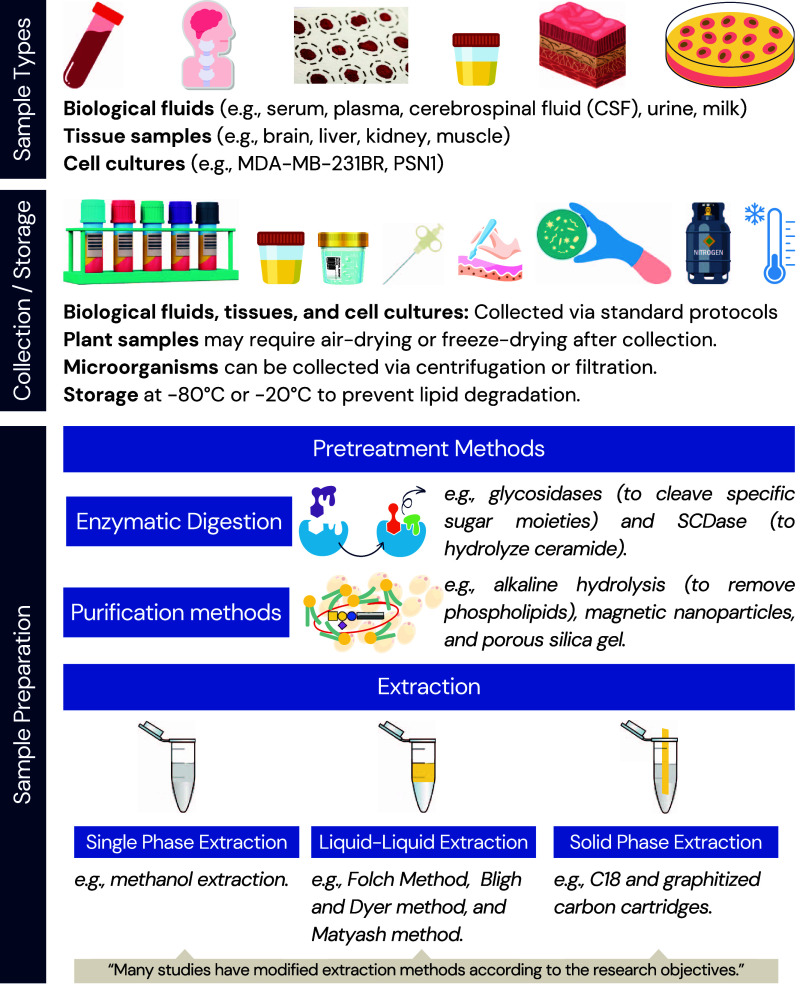
Overview of sample types, collection and storage methods, preparation techniques, extraction strategies, pretreatment, enzymatic digestion, and purification methods for glycosphingolipid analysis. CSF: cerebrospinal fluid; SCDase: sphingolipid ceramide *N*-deacylase.

Another group of advancements and innovations in the analysis of GSLs has focused on overcoming challenges associated with low abundance by developing extra enrichment techniques. One innovative strategy by Wang et al. involves using titanium dioxide (TiO_2_) magnetic nanoparticles (MNPs) for the selective enrichment of neutral GSLs. This method leverages the stronger affinity of TiO_2_ for *cis*-diol structures compared to phosphoric acid, enabling the effective removal of highly abundant phospholipids, which typically hinder GSL detection. Using this approach, researchers achieved approximately 30-fold enrichment of neutral GSLs along with high recovery rates and reproducibility.^48^

From an analytical perspective, it is crucial to note that the extraction method will also determine the detected GSL classes. In other words, while many current extraction techniques are effective for small- to medium-sized GSLs, the information regarding GSLs with long glycan chains remains uncertain, raising the question of whether such species are genuinely absent or simply undetected due to limitations in extraction and analysis methods. For example, in leukocytes, GSLs with glycan chains containing up to 15 sugars, including sialylated and fucosylated species, were successfully extracted using a multistep chloroform–methanol–water protocol, followed by solid-phase extraction (SPE) enrichment.^97^

### 2.3 Enzymatic Digestion

The analysis of intact GSLs can be challenging due to the extensive structural heterogeneity arising from variations in both the glycan headgroup and the ceramide moiety, in combination with their low abundance in biological samples. To address these challenges, a class of analytical strategies has been developed, focusing on the selective digestion of GSLs. This approach enhances analytical sensitivity toward glycan-centered or lyso-GSL-related pathways.^86^ The main idea is to collapse multiple subclasses into a single signal, enabling a more focused analysis of the glycan headgroup or lyso-GSL changes that may be masked in intact GSL analyses.

Among these enzymatic strategies, those based on endoglycoceramidase (EGCase) digestion have become central to GSL-glycomics workflows. EGCase selectively hydrolyzes the linkage between the glycan headgroup and ceramide, thereby ensuring specificity toward GSL-derived glycans. Combinations of EGCase digestion and glycoblotting-assisted sample preparation analysis enabled the reproducibility and accuracy needed for quantitative GSL-glycome profiling.^98,99^

Complementary to GSL-glycome analysis, sphingolipid ceramide *N*-deacylase (SCDase) has been employed as an enzymatic digestion strategy to enable an intermediate-level structural simplification of GSLs. While EGCase cleaves the glycan-ceramide linkage to liberate free oligosaccharides for glycan-centric analysis, SCDase selectively removes the *N*-acyl fatty acid, generating lyso-GSLs that retain the glycan headgroup linked to the sphingosine base.^86^

In addition to enzymatic digestion, chemical strategies are also available for glycan release that can be cost-effective on a large scale. Wang et al. conducted a comparative analysis of chemical approaches for glycan release, demonstrating that an ozonolysis-based method yields the highest glycan release from GSLs under relatively mild reaction conditions. Following this experimental procedure, isotopic labeling with Girard’s reagent P and analysis via HILIC-UV-MS2 were employed to assess the released glycans.^100^

Enzymatic approaches provide advantages for analyzing GSL, but they require high-quality, well-characterized enzymes for reliable results. Enzyme activity can be affected by factors such as storage, purification, and production variability. Studies show that even enzyme preparations with similar purities can exhibit significant differences in catalytic activity, affecting assay results. Thus, maintaining strict quality control and ensuring consistency in reagent sourcing are crucial for effective enzymatic analysis.^101^

After sample preparation, the sample can be either directly infused into the mass spectrometer or subjected to additional separation techniques. A comprehensive overview of direct infusion (DI) MS-based strategies for GSL analysis is given in the recent review by Hořejší and Holčapek.^15^ Although direct infusion MS has many advantages, including rapid and simple injection of the sample into the MS, DI–MS cannot distinguish isomers or isobars easily.^102^ This review emphasizes efficient, accurate, and multitargeted LC–MS-based detection methods for GSL analysis. It highlights the effectiveness of integrating various MS techniques with LC to address challenges in GSL detection.

### 2.4 Creation of Analytical Batches and Sample Types

Along with extracted biological samples, incorporating system-suitability and quality-control (QC) samples into analytical batches is critical for generating reliable analytical data, as extensively reviewed by Broadhurst et al. in LC–MS settings.^103^ In short, two types of blanks, including system-suitability blanks and process (extraction) blanks, a system-suitability sample, and three types of QC samples, including pooled QC, standard reference material (SRM), and long-term reference (LTR), are required, along with biological samples. A system-suitability blank (no injection or solvent-only) should be injected first to verify a clean baseline, followed by a system-suitability sample composed of standards to confirm that the *m*/*z* accuracy, retention-time stability, and peak shape/area meet predefined acceptance criteria. One injection of a system suitability sample at the end of each batch can indicate system performance quality failure before moving to the in-depth, time-consuming data analysis. Pooled QCs, prepared by mixing aliquots from all (or a representative subset of) study samples, should be injected right after a system-suitability sample as a short conditioning series (commonly ∼8 injections) to equilibrate the system. These injections should be excluded from precision calculations. The process blank, prepared by executing the full sample preparation workflow with the biological matrix replaced by an appropriate solvent, should be placed midconditioning to detect systematic contamination and also after the final pooled QC to assess cumulative carryover. It is critical to avoid directly injecting an important biological sample immediately following any blank injection. Instead, a set of pooled quality controls is required to effectively recondition the analytical system before resuming sample analysis. Signals observed in blanks can then be used to flag or remove contaminant features during data cleaning. Pooled QCs should also be injected every 5–10 samples during the analytical block to ensure consistency of the analysis, with two pooled QCs at the start and two at the end as safeguards. Finally, it is recommended to include three SRM injections per batch (e.g., NIST SRM 1950) to support interlaboratory comparability and analyze LTR aliquots (typically up to three times per study) to monitor long-term performance.^103^

### 2.5 Separation Methods in GSL Analysis

Before MS analysis, separation methods, such as LC, ion chromatography, ion mobility, capillary electrophoresis, gas chromatography, and supercritical fluid chromatography, are vital for optimizing intact GSL analysis. Among these techniques, LC coupled with MS is particularly prevalent, especially for polarity-based separations.

The choice of HPLC methods and columns plays a crucial role in the development of the GSL separation method and in enhancing the specificity in complex lipid samples. Hydrophilic interaction chromatography (HILIC) and reversed-phase (RP) chromatography are widely used for polarity-based separation, both employing gradient elution. HILIC utilizes a polar stationary phase, making it ideal for class-level separation based on the glycan headgroup, effectively retaining more polar GSLs. However, it is not capable of performing baseline separation of individual species.^104–106^ An example can be found in a HILIC method that employs a silica hydride-based stationary phase, coupled to TOF-MS, for the identification of low-abundance phospholipids and sphingolipids.^56^ In contrast, RP chromatography uses a nonpolar stationary phase, typically C18, to separate GSLs based on their hydrophobicity. A notable case is the application of ultraperformance liquid chromatography (UPLC) utilizing a C30 RP column, which facilitates the separation of sphingolipids and ceramides. This methodology allows for comprehensive analysis when paired with a hybrid triple quadrupole/ion trap MS system.^61^ Moreover, RP techniques benefit from the Equivalent Carbon Number (ECN) model, which aids in differentiating lipids based on acyl chain length and degree of unsaturation.^104,105,107–109^

Optimizing mobile phase composition is essential for ionization and retention in the LC–MS analysis of GSLs. For instance, the study by Kim et al. uses an aqueous solution with ammonium hydroxide to enhance ganglioside elution on a phenyl column for the separation of gangliosides according to their number of sialic acids and their carbon chain length.^82^ A gradient mobile phase consisting of acetonitrile with ammonium formate was successfully applied in a HILIC column setup, achieving consistent elution profiles for sphingolipid isomers.^56^ In a different approach, mobile phase A consisting of acetonitrile/water and mobile phase B consisting of acetonitrile/isopropanol, both containing 0.1% formic acid and 10 mmol/L ammonium formate, were used to analyze the expression abundance of sphingolipids using UPLC and a C30 column.^61^ The acidic eluent conditions in this study optimize ionization for positive-mode analysis, while in other studies with higher pH eluents, negative mode is more favorable. These methods demonstrate the importance of selecting appropriate columns and mobile phases to enhance the peak resolution, ionization efficiency, and separation efficiency for complex GSL samples.

Innovative approaches, combined with recent advances in LC method development, have significantly expanded the analytical capabilities for GSL studies. For example, a porous graphitized carbon (PGC) nanocolumn combined with RP nano-LC demonstrated high specificity in GSL and glycan separation when paired with an ion trap MS system.^110^ In another study, a multistep workflow was developed for isolating neutral GSLs from human gastric adenocarcinoma, involving alkaline hydrolysis, dialysis, silicic acid chromatography, and ion-exchange chromatography, followed by purification using porous silica particles with a chloroform–methanol gradient. These steps produced a purified product verified by thin-layer chromatography and characterized via LC–MS.^111^ Lastly, two-dimensional liquid chromatography (2D-LC–MS) further enhances GSL analysis by maximizing the separation space through orthogonal and compatible dimensions. For example, a combined HILIC-RP-MS workflow demonstrated the ability to simultaneously separate polar and nonpolar lipids, integrating the benefits of HILIC and RP chromatography. This approach leverages the orthogonal selectivity of HILIC and RP to combine class-specific and lipid species-based separation while maintaining excellent retention time stability and detection limits in the fmol to pmol range.^105,112^ Collectively, these advancements demonstrate the importance of tailored multidimensional chromatography in addressing the complexities involved in GSL analysis.

Ion mobility is another powerful separation method for intact GSL, operating by separating ions based on their size, shape, charge, and apparent surface area in an electric field.^107,108,113,114^ Ion mobility spectrometry (IMS) approaches can be broadly classified into time-dispersive and space-dispersive methods. Time-dispersive systems, including drift tube IMS (DTIMS) and traveling wave IMS (TWIMS), facilitate the separation of GSL ions by analyzing their transit times through a comparable pathway, resulting in the generation of an arrival time spectrum.^114,115^ Conversely, space-dispersive systems such as field asymmetric waveform ion mobility spectrometry (FAIMS) and differential mobility analyzers (DMA) achieve ion separation by directing ions along distinct spatial paths.^116–119^ These distinct ion mobility modes were successfully employed in the structural analysis of GSLs. For instance, TWIMS-MS has enabled profiling of ganglioside expression patterns in fetal brain tissues affected by anencephaly, providing data on 186 previously unknown anencephaly gangliosides, revealing significant differences in polysialylation and glycoform diversity patterns compared to healthy controls.^114^ On the other hand, a FAIMS-based shotgun lipidomics workflow demonstrated enhanced separation of ganglioside classes and charge states, enabling the identification of 117 unique species from porcine brain, accompanied by a class-specific separation.^119^ These ion mobility platforms offer a powerful complementary approach to the comprehensive characterization of complex GSLs.

### 2.6 Mass Spectrometry in GSL Analysis

MS has become an irreplaceable tool in GSL research for offering the sensitivity and specificity needed to resolve complex structures of GSLs.

At the core of any MS-based workflow is the ionization process to transform neutral GSL molecules into gas-phase ions for proper detection and fragmentation. Ionization techniques can be classified as hard and soft ionization techniques. For GSL analysis, soft ionization techniques are preferred to preserve the molecular integrity and allow for intact molecule analysis. Soft ionization techniques include electrospray ionization (ESI), matrix-assisted laser desorption/ionization (MALDI), chemical ionization (CI), atmospheric pressure chemical ionization (APCI), desorption electrospray ionization (DESI), direct analysis in real time (DART), and soft ionization by chemical reaction in transfer (SICRIT).^17,120,121^ Techniques such as DART and SICRIT operate in open-air environments and can ionize samples in all aggregate states. LC–MS with ESI is preferred for GSLs due to its compatibility with their *m*/*z* range (most GSL species detected in the current workflows are between 600 and 3500 *m*/*z*) and support for multiple charge states. In ESI, gangliosides with one sialic acid appear as [M – H]^−^ or [M + H]^+^, while those with two or more sialic acids, like GD1 and GT1, typically form doubly or triply charged ions. FAIMS reveals a correlation between charge states and sialic acid content.^119,122^ Ionization techniques, fragmentation strategies, and labeling approaches for GSL analysis have been comprehensively reviewed by Barrientos and Zhang.^17^

Selection of the appropriate MS instrument is also critical for GSL analysis, particularly for structural characterization. Low-resolution analyzers such as quadrupole, triple quadrupole, and ion traps are effective for targeted quantification but lack the resolution to distinguish species with relatively close *m*/*z* values. In contrast, high-resolution analyzers such as Orbitrap, TOF, and Fourier-transform ion cyclotron resonance (FT-ICR) provide the accuracy and resolution necessary for detailed GSL analysis, making them indispensable for untargeted glycolipidomics^15,17,123–126^ as well as the annotation of new GSL classes.

In GSL analysis, the choice of MS data acquisition mode is strongly driven by the research objective and highly impacts structural coverage, quantification, and annotation. While full-scan MS1 acquisition ensures comprehensive detection of intact GSLs and potential for high-precision quantification, it does not provide structural detail without MS2 spectra. Consequently, data-dependent acquisition (DDA) remains the first choice in GSL research, offering the MS2 spectra essential for resolving glycan head groups and ceramide backbone variations.^127^ For DDA to function effectively in complex GSL samples, especially when dealing with narrow peak widths of UHPLC, it is crucial to carefully balance parameters such as the scan time and the number of MS2 events per cycle. Additionally, there are nonacquisition delays that occur, from the interscan delay, which arises when the instrument transitions from one function to another, and the computational processing by the software, which selects the top ions for fragmentation from the MS1 acquisition. For example, a DDA cycle with a delay time of 31 msec that performs one MS1 and nine MS2 scans can result in as much as 310 msec of nonacquisition delay due to both the interscan delay and the computational tasks, posing a significant issue for narrow chromatographic peaks. To ensure at least 7–8 data points per peak, which is essential for accurate quantification and identification, it is imperative to optimize cycle times.^128^ While data-independent acquisition (DIA) holds promise for broader MS2 coverage, its application in GSL analysis remains very limited.^129,130^

Ionization mode selection is also critical based on the objective of the study. Studies focused on the glycan headgroup of GSLs often use negative ionization mode. For example, in human milk GSL analysis, a HILIC-MS/MS setup operating in negative mode efficiently distinguishes glycan and ceramide compositions by leveraging the ionized state of sialic acid residues, aiding in structural differentiation of ganglioside types.^71^ Similarly, for acute myeloid leukemia cell lines, a PGC-nano-LC–MS/MS approach operated in negative ion mode allowed for high-resolution differentiation of isomeric GSL glycans, effectively resolving glycan heterogeneity associated with cancer cell differentiation markers.^110^ Negative ionization is also beneficial for sulfatide analysis as it produces stable deprotonated [M – H]^−^ ions via ESI.^131^ On the other hand, studies focused on the fatty acid and sphingosine tails of GSLs often use the positive ionization mode. In a study examining gangliosides produced by fungal cell factories, positive-mode LC–MS/MS demonstrated the ability to detect a variety of sphingoid bases with better ionization efficiency and sensitivity in positive mode compared with negative ion mode. This enhancement is particularly important for the characterization of GSLs, as it results from the addition of protons to the secondary amine present in the sphingoid bases.^132^ Similarly, in the examination of GM3 gangliosides in a neurodegeneration model, the positive ionization mode facilitated high-resolution MS2 analysis, enabling precise differentiation of fatty acyl variants. Interestingly, this study showed that upregulated levels of the GM3 ganglioside subclass, with no specification on length and saturation of fatty acyls, are linked to tauopathy.^133^ Additionally, a study on GSLs in fibroblasts from X-adrenoleukodystrophy patients employed positive ionization to monitor very-long-chain fatty acid-containing GSLs, optimizing the detection of GSL subclasses by focusing on the precursor ions of their long-chain sphingosine bases.^65^

Detecting low-abundance GSLs requires sensitivity-focused approaches tailored for trace-level detection. In this case, Orbitrap and TOF-based MS have played a reliable role in the detection of low-concentration GSLs, enabling trace lipid quantification.^56,77^ Such techniques ensure that low-concentration analytes are reliably detected in their intact form and appropriately quantified.

Fragmentation techniques play a pivotal role in GSL annotation, as they generate the structural information needed to identify glycan headgroups, ceramide compositions, and even some isomeric features. In addition, techniques such as the Paternò–Büchi (PB)^48^ reaction and ozone-induced dissociation (OzID)^134^ have proven especially valuable for localizing double-bond positions within the lipid tail. These approaches, extensively reviewed by Hořejší and Holčapek,^15^ generate more informative fragment ions than conventional collision-induced dissociation (CID) and can even enhance ionization efficiency. The choice of the fragmentation method directly influences the depth and confidence of the structural annotation. The annotation approaches will be discussed in detail in Section 3. Figure 4 provides an overview of analytical methods used in recent studies, illustrating the diversity of instruments and approaches applied in contemporary GSL analysis.

**Figure 4:**
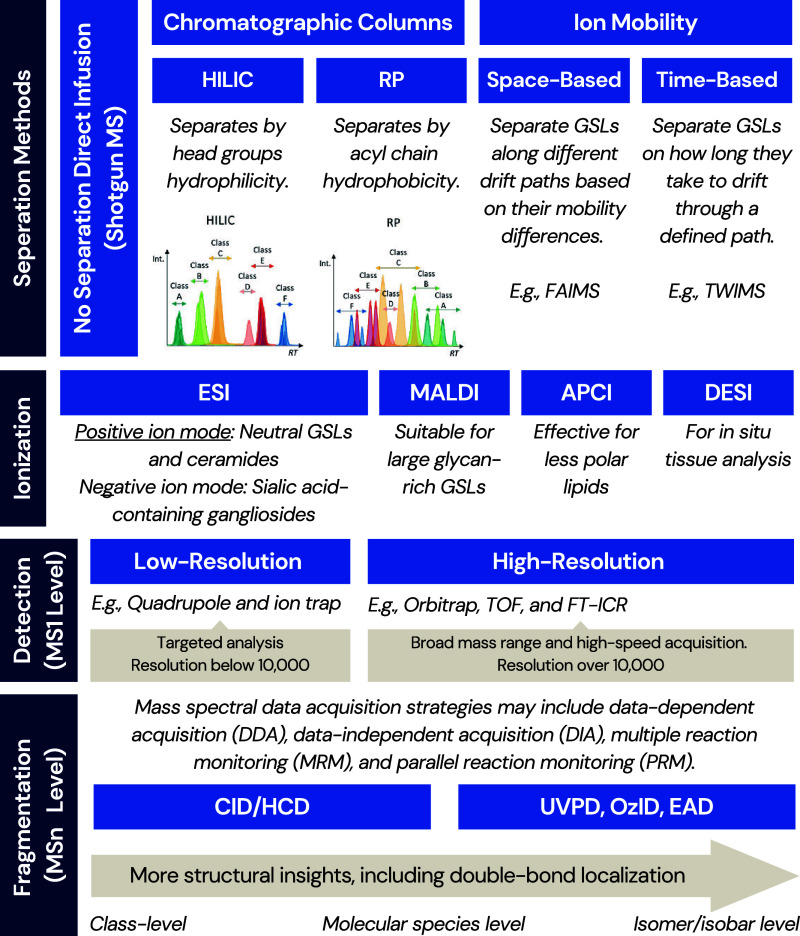
MS-based workflow on separation methods, ionization techniques, MS1-level detection, and fragmentation techniques for GSL analysis. MS: mass spectrometry. HILIC: hydrophilic interaction liquid chromatography, RP: reversed-phase chromatography, SFC: supercritical fluid chromatography, FAIMS: field asymmetric waveform ion mobility spectrometry, ESI: electrospray ionization, MALDI: matrix-assisted laser desorption/ionization, APCI: atmospheric pressure chemical ionization, DESI: desorption electrospray ionization, SICRIT: soft ionization by a chemical reaction in transfer, TOF: time-of-flight, FT-ICR: Fourier-transform ion cyclotron resonance, CID: collision-induced dissociation, HCD: higher-energy collisional dissociation, UVPD: ultraviolet photodissociation, OzID: ozone-induced dissociation, and EAD: electron-activated dissociation.

### 2.7 MS Data Preprocessing

In lipidomics and glycolipidomics, preprocessing choices (feature detection, peak smoothing, grouping, and alignment) play crucial roles in preventing artifacts and misinterpretation. Each compound typically produces multiple MS1 features, including isotopic peaks, various adducts, and in-source fragments, so peak definition must account for these systematically.^135,136^ Selecting an appropriate identification ion and a consistent ion/adduct for quantification is essential for avoiding false positives and false negatives. This is also crucial for the capability and the accuracy of gap-filling approaches.^137^ Orthogonal validation is crucial for accurately defining peaks. In this case, minimal orthogonal validation includes a comparison of positive and negative ionization modes; further orthogonality can come from RP vs HILIC separations, serial extraction, and even alternative ionization/ion-mobility separations, all of which help confirm peak identity and prevent misgrouping.^135,136^ Because GSL isomers often coelute, aggressive smoothing can distort true peak shapes, flatten shoulders, or merge partially coeluting GSL isomers.^135^ Moreover, like neutral lipids, mobile-phase composition can influence adduct formation in neutral GSLs (e.g., promoting formate compared to deprotonated adducts), which directly affects quantitation.^138^

A critical pitfall in GSL data preprocessing involves trihydroxy vs dihydroxy sphingoid bases: dehydration (−18.010565 Da) of trihydroxy ceramide bases during ionization can yield [M + H–H_2_O]^+^ that overlaps the [M + H]^+^ of a dihydroxy species with one additional double bond.^139^ Therefore, the hydroxylation degree should also be confirmed via diagnostic MS2 fragments (e.g., positive-mode sphingoid-backbone ions at *m*/*z* 264/266/282 ^139^) and, when applicable, techniques such as HILIC separation or ion mobility, rather than relying solely on a single mass measurement.

In lipidomics workflows, it is ideal to perform type I and II isotopic corrections prior to quantitative interpretation. In this regard, the type I isotopic effect in GSL analysis is described as the decrease in the proportion of monoisotopic peaks as the number of carbon atoms increases. This effect can be effectively corrected based on the calculated isotopic pattern. Another challenge, the type II overlap, refers to isobaric interference mainly occurring within double-bond series in lipid classes. This interference affecting monoisotopic peaks arises from the M + 2 isotopologue of a species possessing one additional double bond; for example, the M + 2 isotopologue (2 × ^13^C) of [GM3 36:2;O2 + H]^+^ overlaps with the monoisotopic peak of [GM3 36:1;O2 + H]^+^. The mass difference between these isobaric peaks is merely 8.94 mDa. Consequently, when confident separation of these species is not assured (e.g., HILIC and DI) and the MS resolution is not high enough to resolve 8.94 mDa, it is important to evaluate type II overlap.^140,141^

## 3 Key Considerations for MS-Based GSL Annotation

Correct annotation of GSLs is essential for elucidating their roles in cellular processes and disease mechanisms. By leveraging fragmentation techniques of MS-based GSL analysis, it is now possible to explore GSL regulation in much detail, providing deeper structural insights compared to traditional workflows such as antibody detection, thin-layer chromatography, and electrophoresis-based methods.^142^

GSL annotation using MS is dependent on several essential components, including (1) high resolution, (2) high mass accuracy, (3) alignment of retention times across different polarities, (4) analysis of retention time trends through the ECN model, (5) assessment of isomeric and isobaric overlaps, (6) identification of diagnostic fragments, (7) incorporation of orthogonal analysis approaches, (8) biological validation, (9) clarification of the confidence level in annotations, and (10) confirmation using authentic standards along with (11) manual review. Enhancing these factors can reduce GSL misidentifications and prevent the common issue of overannotation. Although practical tools are available for the analysis of typical lipid classes, optimization in these areas remains crucial;^143^ GSL annotation remains particularly challenging due to limited standards, reference spectra, and the absence of fully automated solutions.

The first crucial requirement for a reliable GSL annotation is an acceptable MS resolution, which provides accurate *m*/*z* values. In a HRMS analysis, a five ppm filter is commonly applied at the MS1 level, while a 10 ppm filter is often used for the MS2 level.^144^ Since different mass spectrometers vary in mass accuracy, instrument-specific optimization is essential. Adduct formation and charge states provide additional validation for the GSL classification. Gangliosides, for example, exhibit charge states that vary according to the number of sialic acids that they contain. Species such as GM classes, having a single sialic acid, typically appear as singly charged, while those with multiple sialic acids tend to form more highly charged species. Understanding these charge-state patterns helps ensure correct adduct assignment during MS analysis but also increases the need for higher-resolution MS because compounds with higher charge states tend to overlap more within the five ppm range.

Additional separation parameters, such as retention time trends in chromatography or collision cross section (CCS) compensation voltage values for ion mobility, serve as another key validation factor. In RP-based LC, less polar GSLs elute later, whereas in HILIC-based LC, more polar GSLs elute later. Matching retention times across positive and negative ion modes further strengthens annotation confidence, while ECN models provide additional support for retention time confirmation.^108^

Many misannotations occur due to isomeric and isobaric overlaps. High-resolution MS, isotopic pattern analysis, and fragmentation pattern assessment are essential for distinguishing these closely related species. Although commercial standards for GSLs are limited, they should be used whenever possible. For certain classes, it is necessary to consider the presence of all isomer species at the glycan level, such as GD1a and GD1b, during the validation processes. Additionally, GSL annotations should be consistent with established biological pathways to verify that the identified GSLs align with the anticipated metabolic activities. Manual verification is often necessary, particularly when isomers or isobars are involved, to ensure the accurate identification of GSLs. Ultimately, to maintain transparency and avoid overannotation, it is crucial to report the confidence level of the annotations derived from the analytical workflow.

As illustrated in Figure 5, GSL annotation progresses through a hierarchical framework:

- Sum composition level (molecular formula): At the initial level, GSLs should be identified based on their molecular sum composition, which the *m*/*z* ratio can determine.
- GSL class level (lipid species level): The next level of annotation is to classify GSLs according to their headgroup compositions. At this stage, fragments corresponding to the glycan moieties are used to differentiate GSL classes.
- GSL class and ceramide composition level (molecular lipid species level): Further refinement is accomplished through the examination of the ceramide backbone of GSLs, which differs in its composition of fatty acids and sphingoid bases.
- Isomeric and stereochemical level: The most detailed level of annotation addresses isomeric variations, such as the position of double bonds and the stereochemistry of glycan linkages.

**Figure 5:**
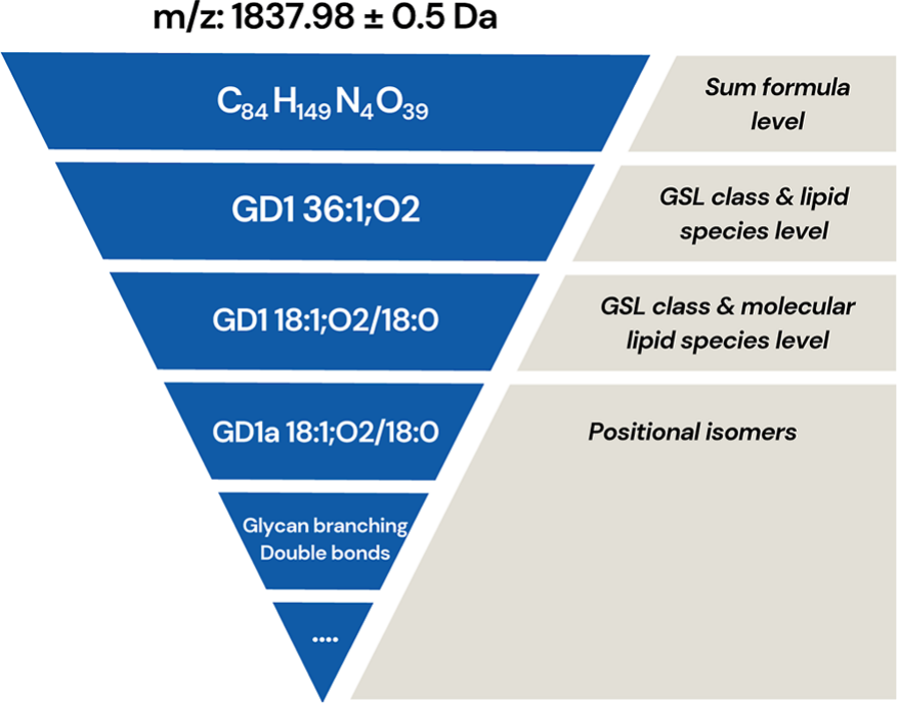
Hierarchical levels of GSL annotation in LC–MS workflows.

Standard workflows, such as RPMS with collision-induced dissociation (CID), often support identification up to the molecular lipid species level, but reaching deeper annotation levels depends on advanced fragmentation or derivatization strategies. Ultimately, the comprehensive annotation of GSLs involves a hierarchical multilevel approach.

### 3.1 Nomenclature of GSL MS2 Fragments

The most widely adopted framework for carbohydrate/glycoconjugate fragmentation nomenclature was introduced by Domon and Costello^145^ and later extended for lipid-backbone information in sphingolipids by Ann and Adams.^146^

In Domon and Costello’s framework, introduced in 1988, fragment ions are categorized based on whether they retain the nonreducing end (A, B, and C ions), which consists of only glycan composition, or the reducing end (X, Y, and Z ions) of the glycan, which also contains lipid information. In addition, in glycosidic bond cleavages, B, C, Y, and Z ions are primarily used to determine carbohydrate sequences, generating stepwise mass losses that correspond to individual monosaccharide units. In positive ion mode, B ions result from protonation and C ions from H-transfer, with characteristic fragments such as B_1_^+^ (*m*/*z* 163) and C_1_^+^ (*m*/*z* 181) from common terminal hexose units like Glu. Y-type ions correspond to neutral losses of monosaccharides (e.g., [M + H–162]^+^), while Z-type ions are more likely to arise from secondary dehydration of Y ions (e.g., [M + H–180]^+^). In negative ion mode, fragmentation begins with deprotonation and involves hydrogen transfer, leading to mass-shifted fragments, for example, the same B_1_^–^ (*m*/*z* 161) and C_1_^–^ (*m*/*z* 179), as well as Y and Z ions, which reflect changes in ion chemistry. A- and X-type ions arise from cross-ring cleavages and provide structural information about linkage positions. A-type ions are more rarely observed in positive ion mode, while X-type ions appear in both modes and often represent neutral losses from the precursor (e.g., [M + H–134]^+^ or [M–H–134]^−^). Comparing glycosidic (B, C, Y, Z) and cross-ring (A, X) fragments across different ionization modes provides complementary insights into sequence and linkage, thereby enhancing the confidence in GSL structural annotation. It is also worth noticing that Y′ (Y ions with a loss of water) ions can share the same *m*/*z* as Z ions.^145^

To account for the complexity of the lipid part of the GSLs, Ann and Adams extended the Domon–Costello nomenclature. Their framework introduces E, G, H, J, K, L, M, O, P, Q, S, T, U, and V ion types.^146,147^ Table 1 and Figure 6 show which fragments each ion type retains.

**Table 1:**
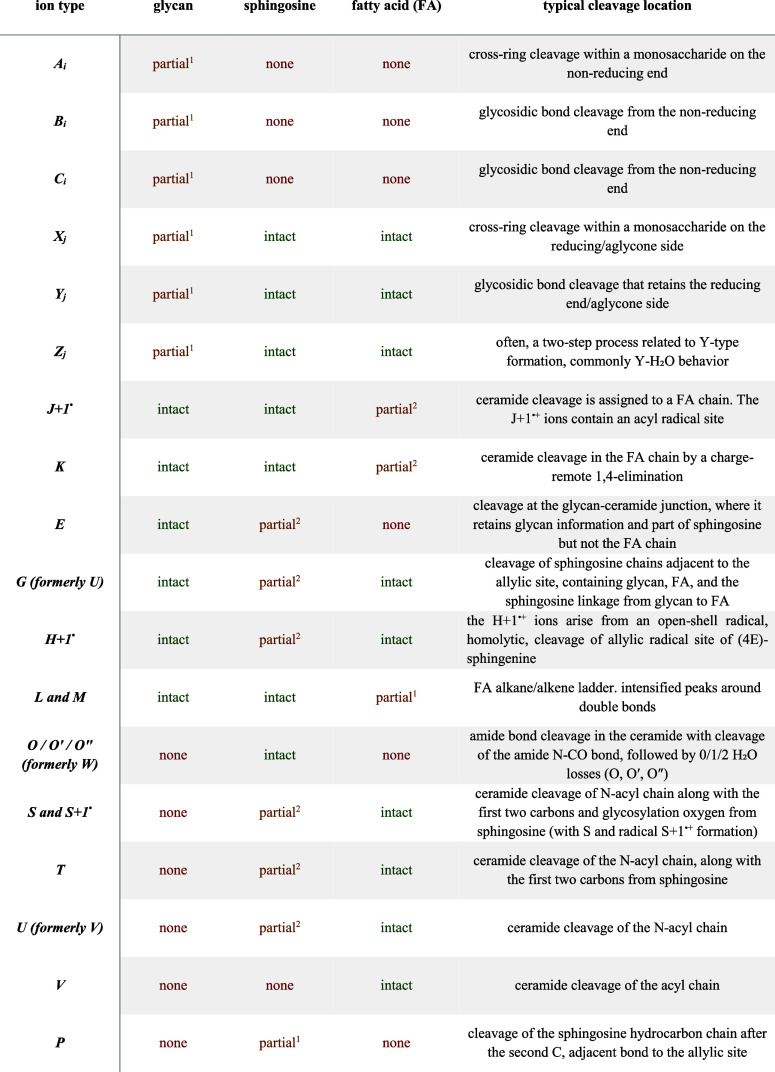
MS2 Fragments Resulting from the Cleavage of GSLs

a The *m*/*z* value is influenced by the chemical composition of the GSLs.

b The *m*/*z* value of this fragment is constant.

**Figure 6:**
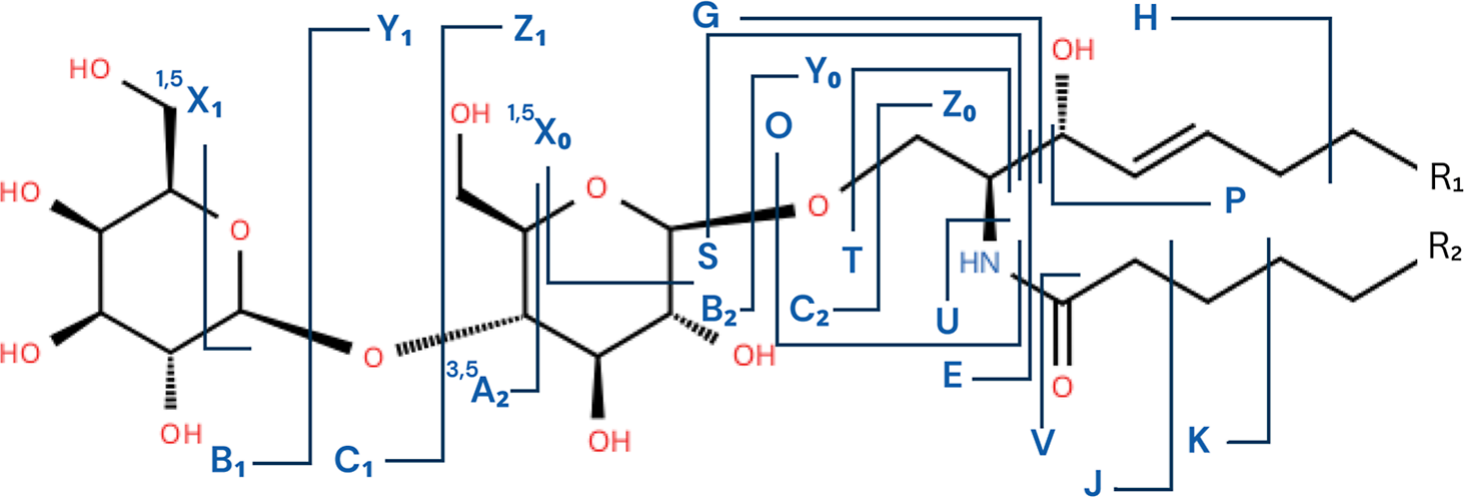
Fragmentation nomenclature used for the glycosphingolipid MS2 spectra.

In practical annotation workflows, a confident GSL structural assignment relies on the combined interpretation of multiple fragment families rather than on any single diagnostic ion. Glycosidic B/C/Y/Z ions are typically used to annotate the glycan sequence and cross-ring ions including A and X refine carbohydrate linkage information Ceramide-specific ions such as O″ or E support assignment of the sphingosine long-chain base and U (formerly V) supports FA assignment. Consistent use of standardized fragment nomenclature facilitates transparent reporting and comparison across studies and reduces the risk of misannotation in complex GSL MS2 data sets.

### 3.2 Diagnostic Fragments

Accurate GSL annotation relies mainly on diagnostic fragment ions that reveal key structural components within GSLs, primarily but not limited to sialic acid, glycan moieties, and long-chain base (LCB) fragments. Hence, generating fragment ions, commonly with CID or higher-energy collisional dissociation (HCD) techniques, is an essential step in GSL structural characterization. Although these techniques are effective for determining the lipid class and species, they usually lack precision for locating double bonds. In lipidomics, strategies such as the use of the PB reaction, epoxidation, and singlet oxygen reaction have expanded structural analysis, enabling double-bond localization. Gas-phase ion activation techniques, such as OzID, metastable atom-activated dissociation (MAD), charge-remote fragmentation (CRF), and radical-directed dissociation (RDD), provide further structural detail. UV photodissociation (UVPD) and electron-activated dissociation (EAD) methods, including electron capture dissociation (ECD), hot ECD, electron-induced dissociation (EID), and electron impact excitation of ions from organics (EIEIO), enable high-resolution structural characterization.^16,17,48,148^

CID and HCD, which utilize neutral gases such as nitrogen, are widely employed in GSL analysis. These techniques often produce glycan-specific fragments in negative ion mode, while ceramide-specific fragments are more frequently observed in positive ion mode. For example, in ganglioside analysis, the loss of sialic acid with an *m*/*z* of 290 is a common and distinguishing marker in negative mode.^82^ Although sialic acid fragments can also appear in positive mode at *m*/*z* 292 as [M + H]^+^, they are less reliable as unique markers due to their low intensity and similarity to fragments from fatty acid 20:1;O2.^48,123^ In addition, negative ionization is also beneficial for sulfatide analysis as it produces diagnostic fragment ions like *m*/*z* 97 for HOSO_3_^–^ and *m*/*z* 259, 257, and 241 for 3-sulfogalactosyl.^131^

Negative mode fragmentation is particularly effective for characterizing glycan head groups. In this case, a study by Wang et al. was able to highly differentiate fragmentation patterns of the glycan part, including glycosylation features and branching.^110^ On the other hand, LCB fragments are typically analyzed in positive mode. For instance, *m*/*z* 264 is a widely recognized marker for 18:1;O2, while *m*/*z* 278 and 292 correspond to 19:1;O2 and 20:1;O2, respectively, under CID conditions.^48,65^ Alternative fragmentation methods such as EAD and UVPD were found to produce an increased number of structurally interesting fragment ions for lipids, enabling the annotation up to the molecular lipid species level and, in some cases, the double-bond position. For GSLs, approximately 10× more fragments can be obtained by both UVPD (shown by Brodbelt and our lab) and EAD (according to measurement of GSL standards in our lab, data not shown) compared to CID, and assignment of glycan class, ceramide, and fatty acid structure in a single fragmentation spectrum can be obtained.^149–151^

## 4 Annotation Strategy Based on Analytical Capabilities

Accurate GSL annotation depends on the resolving power of the analytical workflow including chromatographic separation, mass resolution, and MS2 evidence. Therefore, structural assignments should follow a hierarchical decision logic that considers the analytical platform’s limitations. Lipidomics guidelines also emphasize reporting structures only at a level supported by experimental evidence to prevent overinterpretation of MS data. In addition, annotated species should be detected above the instrumental sensitivity threshold and supported by reproducible signal intensity to ensure that the reported features represent genuine analytes rather than noise-level signals.

A practical approach to confident annotation of GSLs can be based on the proven resolving power of the analytical workflow, as shown in Figure 7 and explained in Table 2. This decision-tree framework evaluates two structural dimensions: the glycan headgroup (G) and the lipid tail (L). A proper annotation, with attention paid to the confidence level, can be a combination of the most confident nomenclature derived from each structural dimension separately.

**Figure 7:**
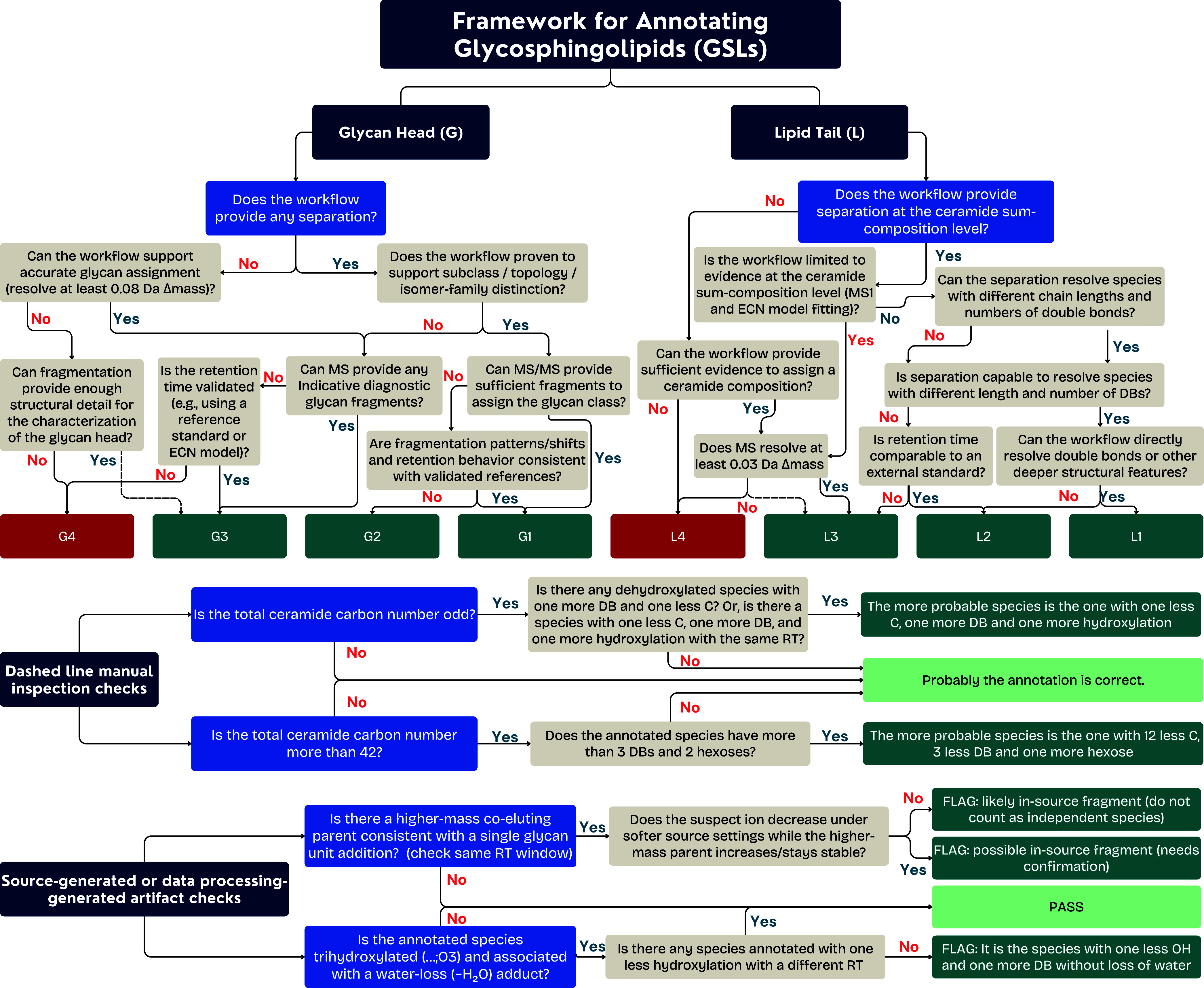
Decision-tree framework for glycosphingolipid annotation based on the demonstrated capabilities of the analytical workflow. The scheme evaluates the level of structural evidence separately for the glycan headgroup (G) and ceramide moiety (L), considering factors such as chromatographic separation, mass resolution, and availability of diagnostic MS2 fragments. Based on this evidence, annotations are assigned within a hierarchical system ranging from low to high structural resolution. For the glycan headgroup, levels include G4 (glycan unresolved), G3 (glycan sum composition level; e.g., Hex3HexNAc1NeuAc1), G2 (glycan class level; e.g., GM1, GM3, Gb3, LacCer), and G1 (glycan subclass level; e.g., GM1a, GD1a). For the ceramide moiety, levels range from L4 (ceramide unresolved) to L3 (species level; e.g., 36:2;O2), L2 (chain-resolved ceramide level; e.g., 18:1;O2/18:1), and L1 (more detailed structural characterization such as double-bond-localized ceramide level; e.g., 18:1­(Δ4);OH,3OH/18:1­(Δ9)). This framework guides the reporting of GSL structures at a level supported by the analytical data and helps prevent overannotation beyond the method’s resolving power.

**Table 2:** Hierarchical Annotation Levels for GSLs Based on the Degree of Structural Information Available for the Glycan Headgroup (G) and Ceramide Tail (L), Illustrating How Combinations of G and L Levels Define the Confidence and Detail of Reported GSL Structures

level		code	annotation level title	example
unknown	head group type	G3 + L4	glycan sum composition + unresolved tail	**Hex3HexNAc1NeuAc1Cer**
		G2 + L4	glycan class + unresolved tail	**GM1**
		G1 + L4	glycan subclass + unresolved tail	**GM1a**
partially assigned GSL structure	lipid species	G3 + L3	GSL sum composition	**Hex3HexNAc1NeuAc1Cer 36:2;O2**
	sum composition	G2 + L3	glycan class + ceramide species level	**GM1 36:2;O2**
	glycan composition	G1 + L3	glycan subclass + ceramide species level	**GM1a 36:2;O2**
	molecular species	G3 + L2	sum composition + chain-resolved ceramide	**Hex3HexNAc1NeuAc1Cer 18:1;O2/18:1**
	FA composition	G3 + L1	sum composition + lipid full structure level	**Hex3HexNAc1NeuAc1Cer 18:1(Δ4);1OH,3OH/18:1(Δ9)**
assumed GSL structure	molecular species	G2 + L2	glycan class + chain resolved ceramide GSL level	**GM1 18:1;O2/18:1**
	glycan composition	G1 + L2	glycan subclass + chain resolved ceramide	**GM1a 18:1;O2/18:1**
	double bond position and high structural characterization	G2 + L1	glycan class + lipid full structure level	**GM1 18:1(Δ4);1OH,3OH/24:1(Δ15)**
		G1 + L1	highest structural GSL characterization level	**GM1a 18:1(Δ4);1OH,3OH/24:1(Δ15)**

Attention to workflow capabilities can help avoid overannotation. For example, if the analytical method does not provide a clear separation of glycan subclasses of isomers (e.g., GM1a, GM1b), the annotation should remain at the class level that is experimentally distinguishable. Even when a GM1a standard is available for comparison, a study without sufficient subclass separation should avoid reporting the specific isomer GM1a in complex biological samples, as coeluting glycan variants may lead to misleading biological interpretation.

Additionally, to address common misannotations, the workflow incorporates biological plausibility checks and artifact-detection procedures. Ceramides exhibiting unusual carbon chain lengths, improbable double-bond conformations, or discrepancies with established biosynthetic principles are flagged as potential misannotations. For example, low-resolution MS without chromatographic separation and sufficient fragmentation cannot confidently distinguish between species such as GM2 46:3;O2 and GM1 34:0;O2, which differ by approximately 0.088 Da as shown in Figure 7. Similarly, even when chromatographic separation is available, small mass differences between possible elemental compositions (e.g., GM3 35:0;O2 vs GM3 34:1;O3, Δ*m* ≈ 0.0364 Da as in Figure 7) may remain unresolved if the separation resolving power is insufficient to confidently support the ECN model and retention time validation.

It is worth noting that the criteria on the workflow can be flexible regarding study objectives and target organisms. According to research on human samples, the predominant fatty acyl moieties in the ceramides of mammalian GSLs are typically 16-, 18-, 20-, and, in rare occurrences, 24-carbon fatty acyl. The most common sphingosine base also contains 18 carbons.^96^. Consequently, the identification of ceramides with over 42 carbon atoms is unlikely, particularly when detected using low-resolution MS. Similarly, the presence of odd-chain ceramides is possible but uncommon, necessitating manual examination, particularly in low-resolution workflows.^6^ Nonetheless, variations may occur depending on the target organism; thus, we strongly recommend establishing thresholds and filters based on incorporating biological intelligence and pathway logic.

Moreover, the workflow includes a verification step for in-source fragmentation artifacts such as lower-mass species resulting from glycan loss within the ion source, which could mistakenly be interpreted as separate GSL species. Additionally, as previously noted, the loss of water from a trihydroxylated species should be handled with greater caution. Collectively, these measures contribute to a structured approach that minimizes overannotation and enhances the reliability of reported GSL structures.

### 4.1 Automated GSL Annotation Approaches

Advances in MS-based fragmentation interpretation have positively impacted the fields of lipidomics and glycolipidomics by enabling more detailed structural elucidation. However, GSL annotation remains a considerable challenge due to limited software capabilities and the absence of comprehensive databases. Accordingly, GSL annotation is conducted manually to a large extent, which is complex and therefore error-prone due to the diverse fragmentation pattern. A major problem for GSL annotation is obviously the computational complexity and filtering for false-positives. Therefore, independent validation strategies for intact GSL analysis such as enzymatic release and parallel glycan annotation of GSL or GSL derivatization by permethylation can help to validate intact GSL analysis and identification of the correct GSL in a sample.^152^ In general, intact GSL annotation relies on two primary strategies: spectral library matching and rule-based annotation. Each method has its strengths and limitations, and its effectiveness depends on the availability of reference data, accessible commercial standards, and the complexity of the GSL class being analyzed. While spectral library matching provides rapid and automated identification, its reliability is limited by the completeness, accuracy, and coverage of the database across instruments. Rule-based and in silico annotation, in contrast, allows the identification of novel GSL species and is particularly useful for underrepresented classes, but developing rules for unsupported GSL classes requires expert knowledge of fragmentation.

### 4.2 Library-Based Annotation Tools for GSL Analysis

Spectral library matching is a well-established technique that enables the rapid and automated identification of molecules. This method involves comparing MS2 spectra with a database of reference spectra, typically sourced from authentic standards or generated through computational in silico fragmentation. A key benefit of spectral matching is its provision of fast sample analysis and scoring metrics that offer a measure of the confidence in the annotations.

The effectiveness of the spectral library matching approach is closely linked to the quality and coverage of the reference database utilized. Several factors can impact the accuracy of spectral library matching, including variability in fragmentation spectra across different MS instruments, discrepancies in collision energies and fragmentation techniques, the reliability of annotations within the reference spectral library, and variations in the experimental conditions across different laboratories. Ideally, spectral entries should be derived using the same parameters as those applied during the sample measurements to ensure consistent and reliable results. Another challenge is the limited availability of high-quality public spectral libraries. Many in-house libraries are not shared, and vendor-specific libraries often require paid licenses, limiting their accessibility. Additionally, no standardized guidelines currently exist for spectral library construction, further complicating the generation of universally applicable reference databases.

Vendor-specific as well as open-source software solutions are available for library-based annotation. Among open-source solutions, mzmine is widely used for data processing and annotation, offering a flexible platform for peak picking and data analysis in LC–MS data sets. Among commercial solutions, LipidSearch provides advanced capabilities for lipid identification and annotation, particularly for detailed lipid species analysis.^58,67,153^

Several databases also support GSL annotation, providing reference frameworks for structural characterization. Notable resources include LIPID MAPS [e.g., LMSD],^48,67,153^ HMDB (Human Metabolome Database),^48,63,154^ METLIN,^154^ and LipidBlast.^62^ However, in contrast to other lipid classes, the enormous GSL search space is not well covered by these global resources.

The software landscape for GSL annotation remains limited compared to tools for common lipid classes such as phospholipids, with resources like AdipoAtlas and Lipid Maps Tools Guide providing little support for GSL analysis.^155,156^ Moreover, many solutions, such as MSDial, mzmine, LipidXplorer, and Compound Discoverer, which are used for bulk lipids, lack compatibility with specialized techniques like shotgun FAIMS, as their peak-picking algorithms are optimized for LC–MS rather than continuous signals from shotgun methods.^130,157,158^ In conclusion, limited entries and incomplete libraries for GSL classes, chain lengths, and adducts hinder accurate annotation, highlighting the need for more tailored tools.

A recent study by Giang Vo et al. presents a major step forward in GSL spectral library development through the creation of the 4D-sialoGSL standards library, which integrates four orthogonal dimensions of mass spectrometric information: *m*/*z*, retention time, collision cross section (CCS), and MS2 spectra acquired using the parallel accumulation-serial fragmentation (PASEF) method. This library was generated by using both commercially available GSL standards and porcine brain extracts, enabling the structural annotation of 226 gangliosides. The authors addressed one of the major limitations in GSL annotation, the absence of experimental spectra in existing databases, by curating experimental MS2 data in both positive and negative modes and manually validating structures based on diagnostic fragment ions. These spectra were used to build a searchable reference resource tailored for RP-LC-TIMS-PASEF analysis.^159^

### 4.3 Decision Rule-Based and In Silico Annotation Tools for GSL Analysis

Recent developments in rule-based and in silico annotation have significantly advanced the structural identification of GSLs and other complex lipid classes in LC–MS/MS workflows. While tools like LipidBlast provide broad in silico MS2 spectral libraries across 26 lipid classes, including glycolipids, their utility lies primarily in spectral matching. In this case, LipidBlast’s flexible template system enables rapid generation of libraries for novel lipid classes, facilitating high-throughput identification of previously uncharacterized compounds.^160,161^

In contrast, rule-based annotation offers an advantageous alternative, especially when spectral libraries are sparse or unavailable. Tools like LipidMatch and the lipid data analyzer (LDA) enable lipid identification by analyzing fragmentation spectra using established decision rules rather than relying solely on spectral similarity. LipidMatch supports user-modifiable rules for validating fragment ions,^162^ and LDA enhances rule-based workflows by applying subclass-specific fragmentation rules to facilitate high-throughput annotation of diverse GSL classes, including the application to LC–MS and FAIMS separation for direct intact GSL analysis.^163–165^ In cases where permethylation in combination with direct infusion is performed, DANGO was recently introduced as a decision-rule-based approach for the annotation of intact GSLs.^152^

Rule-based methods are particularly well-suited to handling the high variability of MS2 spectra across different instruments and fragmentation techniques. Their inherent flexibility allows customization and incorporation of platform-specific experimental data, resulting in improved robustness and adaptability. Diagnostic ions used in GSL annotation are selected based on predictable fragmentation behavior; for example, in negative ion mode, ions such as [M–H_2_O]^−^, [Hex–H_2_O]^−^ (*m*/*z* 161), and [HexNAc–H_2_O]^−^ (*m*/*z* 204) are indicative of glycan headgroups. In positive ion mode, LCB fragments, such as *m*/*z* 264 (18:1) and *m*/*z* 278 (19:1), provide information about backbone composition and unsaturation. Together, these features, combined with established fragmentation logic for specific GSL classes that can be applied even in the absence of commercial standards, make decision-rule-based analysis particularly well suited for automated GSL annotation.

#### 4.3.1 Decision-Rule-Based Approaches for GSL Classes without Authentic Standards: GIPCs as a Case Study Using LDA

The absence of commercially available authentic standards remains a fundamental bottleneck in GSL analysis, rendering purely spectral-library-based identification approaches insufficient for many lipid classes. Decision-rule-based strategies address this limitation by using predefined, chemically informed fragmentation and retention rules to deduce the lipid class and structural features without relying on available reference spectra.

Glycosyl inositol phosphoceramides (GIPCs) exemplify this challenge. As the dominant sphingolipids in plant and algal membranes, GIPCs are ubiquitous and biologically essential, yet no authentic standards exist.^166,167^ Their annotation, therefore, depends on plant-specific fragmentation rules rather than library matching. Within the lipid data analyzer (LDA), GIPC identification relies on mandatory diagnostic fragments of the glycosylated inositol phosphate headgroup, most notably [IP–H_2_O]^−^ and [IP]^−^ (*m*/*z* 241 and 259), supplemented by rules capturing higher-order GIPC series and glycan branching isomers.^168^

These constraints enable reliable GIPC detection across diverse plant matrices while minimizing ambiguity from isobaric species. To avoid false annotations, LDA-based rule validation is embedded within a multilayered verification framework. Annotation begins with MS1 feature detection, isotopic deconvolution, and consolidation of denoised MS2 spectra, followed by hierarchical decision-rule evaluation for headgroup, chain composition, and positional information. In parallel, LDA incorporates retention time prediction based on nonlinear ECN models, ensuring chromatographic consistency of annotated species. Importantly, false positives are further reduced by orthogonal retention time matching between positive- and negative-ionization modes, providing an additional acquisition-independent validation layer beyond fragmentation rules alone. Fragmentation rules in LDA are organized modularly, allowing stringent filtering at the headgroup level before evaluating acyl-chain combinations and sn-position information.^169^ Class-specific constraints, including chain number and hydroxylation ranges, are used to limit combinatorial complexity arising from structural heterogeneity. Together with interactive rule adjustment via a graphical user interface, this framework allows expert-guided optimization of annotation criteria for complex GSL subclasses.^169^ Overall, the GIPC case illustrates how decision-rule-based annotation combined with retention behavior, ECN modeling, and polarity-consistent RT matching enables the confident identification of GSL classes that are inaccessible to conventional library-driven workflows.

## 5 GSL Quantification

Quantifying GSLs is a critical yet challenging task in lipidomics aimed at determining their relative or absolute levels in complex biological samples. This process requires a well-validated workflow, including the careful use of internal standards (ISs), but is limited by the vast diversity of lipid species, lack of ISs, and variability across laboratories and instruments.^16,46^

Absolute quantification, relative quantification, and semiquantitative approaches are used to quantify GSLs, each with its own strengths and limitations. Absolute quantification offers the highest precision due to using authentic standards, but its application in quantitative GSL analysis is limited by the availability of isopure and labeled standards for the vast range of GSL species.^46,100,170^ In contrast, relative quantification, which compares peak areas or signal intensities across samples, is more practical for large-scale studies and is widely used in the quantification of GSLs.^100,123^ Semiquantitative analysis in GSL analysis, as demonstrated by Maekawa et al., also provides a practical alternative when authentic standards are unavailable. In this method, estimation of concentration changes across samples was performed by measuring the peak areas of targeted lipids using LC–MS/MS with selected reaction monitoring (SRM).^60^

A range of ISs has been developed to support the quantification of GSLs, but their availability remains a key limitation. Ideally, isotope-labeled ISs that closely match the analyte in structure and ionization behavior should be used to account for matrix effects and variability in the MS response. However, the synthesis and commercial availability of such standards are limited. The review study conducted by Hořejší et al. provides an extensive list of commercially available ISs corresponding to various GSL subclasses, such as GalCer, GlcCer, LacCer, GM1, and GM3, as well as their isotopically labeled counterparts. This compilation stands as a significant resource for researchers endeavoring to develop effective quantification workflows.^16^ In addition, the review by Wang et al. outlines key principles and guidelines for IS selection.^171^ Also, Myers et al. provide important recommendations on data handling and interpretation in quantitative lipidomics.^172^ Collectively, these references form a strong foundation for researchers aiming to establish reliable GSL quantification workflows.

Accurate quantification of GSLs requires proper normalization to reduce variability caused by factors such as sample-to-sample differences, extraction methods, and instrument performance. This normalization is essential for preserving the biological relevance of the data and ensuring that the observed variations truly reflect the biological differences. Normalization techniques can be categorized into three main types: biology-based approaches, IS-based methods, and data-driven strategies.

Biological normalization methods typically adjust lipid abundances using sample-specific biological metrics such as protein content^58^ and dry tissue weight.^163^ These parameters are straightforward to obtain and are commonly used in GSL studies. However, their reliability may be limited in heterogeneous sample sets, where factors such as cell morphology and aggregation can introduce additional variability and significant errors.^173^

Using ISs is the most established method for correcting extraction and ionization variability in GSL analysis.^56,82,144,163^ Isotopically labeled analogs are often preferred due to their strong physicochemical similarity to the target analytes. This similarity helps to ensure reliable adjustments for extraction efficiency and variations in ionization. When such labeled standards are not available, synthetic GSL analogs, odd-chain variants, or chemically similar compounds can be used as suitable alternatives.^132^ However, caution is required; endogenous odd-chain GSLs have been reported,^48^ and using odd-chain GSLs as ISs may compromise the accuracy of quantification.

In cases where ISs or biological parameters are unavailable or where unwanted artifacts are introduced, data-driven normalization provides an alternative. Approaches such as normalization to the sum of GSL intensities^70^ and probabilistic quotient normalization^173^ can be used as data-driven normalization. Notably, in data sets with substantial differences between sample groups, data-driven normalization may outperform biology-based methods.^173^

In addition to normalization, other critical data processing steps must be addressed to ensure robust quantification. These include data filtering, handling missing values, transformation, and scaling. Furthermore, method validation parameters, such as the lower limit of quantification (LLOQ), upper limit of quantification (ULOQ), reproducibility, and linearity range, should be established prior to sample analysis for each batch.

Based on prior studies and analytical best practices, the following stepwise workflow is recommended for GSL quantification:

1. Separation-specific preprocessing should be carried out, including peak detection, deconvolution, retention time or CCS alignment, feature matching/grouping, and, where necessary, batch correction. In addition, QC-based evaluation using pooled QC samples and process blank should be incorporated to assess signal stability and remove background features.^103^
2. Features exhibiting signal intensities beneath the LLOQ or falling outside the designated linearity range and ULOQ ought to be either flagged for attention or discarded from the analysis.
3. Variables characterized by a significant proportion of missing data or values below the LLOQ across all analyzed samples, specifically those exceeding 20%, should be removed from the data set. This is based on the 80% rule introduced by Bijlsma et al.^174^
4. For the remaining missing values, the nature of missingness should guide the selection of the imputation method. Where values are missing completely at random (MCAR) or at random (MAR), imputation using Random Forest (RF) is advised. For values likely missing not at random (MNAR), such as those below detection limits, quantile regression imputation for left-censored data (QRILC) is recommended.^175^ Other imputation strategies reported in the literature include half-minimum, *k*-nearest neighbors (kNN), and the use of a small positive constant (e.g., 0.00001 or zero).^175,176^

5. If appropriate, IS-based normalization should be performed as described previously.
6. Biology-based normalization, using parameters such as protein content or tissue weight, should be applied if relevant and available.
7. In situations where IS and biology-driven normalization techniques are either inaccessible or lead to artifacts, it is advisable to turn to data-driven normalization methods. Options such as probabilistic quotient normalization or normalization based on the sum of GSL intensities should be utilized in place of steps 5 and 6.^70,173^

8. Outliers should be detected and flagged using appropriate statistical techniques, such as the robust regression followed by outlier identification (ROUT) method in GraphPad^69^ or Grubbs’s test.^59,82^

9. In GSL analysis, data often exhibit a wide dynamic range and skewed distributions. Data transformation should then be applied to stabilize variance and reduce skewness. The square root transformation is suitable for data sets with many low values or zeros (zero-inflated data sets),^57^ while log transformation is preferred where zero values are absent.^154^
10. Finally, data scaling should be conducted to prepare the data set for multivariate statistical analysis. Pareto scaling may be applied to reduce the influence of highly abundant lipids while preserving the overall data structure.^57,177^ Alternatively, autoscaling can be used to ensure equal weighting of all variables. The review by van den Berg et al. compares goals, advantages, and disadvantages of each scaling approach extensively.^177^

## 6 Conclusion and Future Perspectives

Building on recent advances in GSL analysis, this review offers a fresh perspective on GSLs and how we interpret their roles. We refer to them as whispers, not only because they are low in abundance and difficult to detect but also because their signals are subtle, ambiguous, and easily misunderstood. Also, like whispers, they require careful, attentive listening to uncover valuable biological information through them to avoid distorted conclusions and spreading analytical “rumors” into the scientific literature. In this discussion, we explore three critical questions that can guide the next phase of GSL research:

### 6.1 Are We Annotating What We Think We Are Annotating?

MS-based annotation of GSLs has seen remarkable progress, yet a central question always remains in an analytical context: “Are we truly annotating what we think we are?” Despite a growing toolbox of high-resolution instruments, spectral libraries, and algorithmic and in silico annotation pipelines, GSL annotation workflows continue to struggle with ambiguity, overannotation, and inadequate standardization, problems amplified by their extreme structural diversity. GSLs are among the most structurally complex biomolecules, consisting of a ceramide backbone and an array of glycan headgroups. As shown in the biosynthesis pathways and diversity diagrams included in this review, these structures are built modularly, using enzymatic “LEGO bricks” that generate a massive number of possible isomeric and isobaric species. This complexity is both a biological asset and an analytical burden. A major limitation arises from the reliance on classical classification of GSLs, such as GD1a, GM1, and GT1b, which were designed for the TLC-era workflows that may not reflect actual molecular resolution.

In many modern LC–MS workflows for intact GSL analysis, no class-specific separation is achieved before annotation. Yet, software and databases still assign shorthand labels (e.g., GD1a) based on assumptions rather than actual class-level discrimination. As a result, overannotation is common, especially in workflows where diagnostic fragments may be weak and the separation between isomers such as GD1a and GD1b is not confirmed. In this example, the presence of a fragment signifying two sialic acids can be annotated as GD1b, yet without class-specific separation, it is a mixture of GD1a and GD1b. Overannotating a peak as a biologically specific ganglioside subclass when only a general sialylated structure has been observed introduces the risk of false mechanistic conclusions in downstream biological interpretations.

To address overannotation problems for intact GSL analysis, a nomenclature reflecting the experimental-derived evidence level is essential not only for improving data integrity but also for aligning annotation practices with the analytical limitations of each method. Fragmentation patterns in CID or HCD rarely provide enough detail to distinguish between isomers without prior chromatographic separation or orthogonal validation such as ion mobility spectrometry or glycosidic linkage analysis.

Additionally, in-source fragmentation must be considered as a source of misannotation. For instance, the increased signal intensity of LacCer species in some studies may not indicate true biological upregulation but rather in-source degradation of more complex GSLs, such as gangliosides, during ionization. Without chromatographic separation or dedicated in-source fragmentation control, such fragments may be misassigned as the primary species. This not only inflates subclass diversity in the data set but also propagates errors in downstream biological interpretation. Therefore, annotations based on in-source fragments should be flagged as tentative unless verified through a retention time comparison, MS2 confirmation, or orthogonal techniques.

In summary, the move toward sum-composition-based annotation with explicit confidence levels is a methodological necessity. It acknowledges the complexity of GSLs, while respecting the resolution limits of analytical techniques and methods and ensuring that biological interpretations are grounded in the actual capabilities of the analytical workflow. Orthogonal methods should always be added, and standards should be added whenever possible.

### 6.2 Is There Really a Biological Relevance, or Are We Introducing Bias through Preprocessing?

As glycosphingolipidomics workflows become more automated, a critical question must be asked: “Are the biological patterns we observe in GSL data truly meaningful, or are they shaped, if not distorted, by our data acquisition and processing choices?” While the analytical technologies behind GSL quantification have advanced, the interpretation of the results remains deeply sensitive to normalization, imputation, and preprocessing strategies. Without careful scrutiny, these steps may not correct for bias; they may create it. The most reliable method for MS-based quantification is having an isotope dilution, which also involves matrix-matched multilevel external calibration alongside internal standardization, and for omics analyses, it necessitates a significant quantity of stable-isotope-labeled external and internal standards.^112^

GSLs are highly responsive to the cellular context, with biosynthesis tightly linked to metabolic, immune, and developmental signaling. However, in many studies, conclusions about GSL class alterations are drawn from relative quantification workflows that use simplified normalization strategies (e.g., normalization to sum or the total ion chromatogram (TIC)) or global signal scaling without confirming whether these changes reflect actual biological shifts or computational artifacts. For example, an apparent increase in one GSL class (e.g., GM3) may not be due to upregulation in biosynthesis but rather to a drop in another class or inaccurate normalization, especially if data-driven normalization (e.g., PQN or total ion current scaling) was applied without validating its assumptions.

This issue becomes more pronounced when biological samples exhibit large intergroup variability, as in case-control designs or cancer cell line panels. Studies have shown that protein content and cell number are frequently used for biology-based normalization. Still, they do not always correlate with the total lipid content, especially in heterogeneous samples with differences in morphology, adherence, or growth phase. In such cases, normalization to protein content may introduce bias rather than eliminate it, and relative comparisons can result in false covariation of GSL subclasses that were not biologically linked.

Even IS-based normalization is not immune to bias. A major source of error is ion suppression or ion enhancement, which arises when the ionization efficiency of the IS differs from that of the endogenous analyte due to matrix effects. For example, in class-based separation workflows, if a deuterated GM3 IS coelutes with highly abundant GM3 species that strongly ionize gangliosides, its signal may be suppressed. This suppression can vary with the level of GM3 expression across sample groups, potentially leading to over- or underestimation of the relative abundance of other endogenous GSLs. Conversely, suppose that the IS elutes in a relatively clean region of the chromatogram and undergoes ion enhancement. In that case, it may artificially reduce the calculated analyte levels by lowering the intensity of the IS signal. These effects can differ significantly across samples, depending on coelution patterns and overall matrix composition, making IS-based normalization less reliable in untargeted or structurally diverse data sets.

To determine whether observed GSL changes are biologically meaningful or normalization artifacts, studies must apply pathway logic and ask whether the changes fit the known GSL biosynthesis networks. For example, an increase in GM1b should be evaluated alongside the abundance of upstream intermediates such as GM3 and GM2, as well as the core precursor LacCer, to determine whether the change reflects a genuine biosynthetic shift at a specific point. If coregulation of precursors and products is absent, or if only one end-point GSL class increases with no intermediates, the signal may reflect a processing artifact. Furthermore, if GSL classes with entirely different headgroup architectures (e.g., gangliosides and globosides) coincrease in an unrelated manner, this can suggest either poor subclass resolution or inappropriate normalization scaling.

Compounding the issue is that studies often lack transparency in how missing values are handled. A GSL species that is missing in 40% of samples but imputed (e.g., with half-minimum (HM)) may appear “upregulated” in cases, not due to fundamental expression changes but due to the effect of imputation combined with normalization. Without clearly stating the imputation method and its rationale, downstream statistics, such as fold change or p value, lose interpretability. The use of quantile regression imputation for left-censored data (QRILC) has been recommended for MNAR-type missingness common to low-abundance GSLs. Yet, many studies continue to use simpler but less appropriate methods like zero or half-minimum imputation, which artificially compress variability.

- To ensure that biological conclusions are not simply artifacts of the data workflow, a few practices should be adopted:
- Track and report missing value percentages per feature and clarify the imputation method and rationale.
- Use normalization approaches tailored to the study design, IS-based for targeted quantification, and only carefully chosen data-driven methods (like PQN on structural lipid classes) when IS or biology-based normalization is unavailable.
- Evaluate the covariation of features using known biosynthetic relationships. If downstream GSLs increase without corresponding changes in their precursors, reassess normalization and signal reliability.
- Avoid overannotation and forced subclass assignments, particularly in nontargeted workflows with poor isomeric resolution.

Lastly, biological validation must accompany computational inference. Observed changes in GSLs must be linked to enzymatic pathways, transcriptional data, or a phenotypic indicator, particularly when the magnitude of the change is considerable. Without such a triangulation, there is a danger of projecting biological significance onto what may be preprocessing noise.

In short, while GSLs are biologically powerful molecules, they are analytically fragile ones. Preprocessing must not only be rigorous but also be biologically aware, hypothesis-informed, and transparently reported.

### 6.3 What Should We Do Next? A Path toward a More Meaningful GSL Research

The previous discussion highlights two significant challenges in glycosphingolipid (GSL) research. First, there is a gap between the annotations we have and the actual structural evidence. Second, fluctuations in GSL levels may result more from data processing issues than from true biological changes. These challenges go beyond technical details; they undermine the credibility of GSLs as both biomarkers and therapeutic targets. This, in turn, obstructs our ability to fully understand lipid-mediated cellular processes. To advance the field, it is essential to implement a series of deliberate, incremental, and innovative modifications across technological, computational, and biological dimensions.

#### 6.3.1 Modernizing Instrumentation and Separation Strategies

Most current GSL workflows still rely on one-dimensional LC–MS platforms, often with limited isomeric resolution. However, given the structural complexity of GSLs, especially the presence of numerous regioisomers and isobaric species, multidimensional or multistage separation techniques should be more widely adopted. 2D-LC, which combines orthogonal separation mechanisms (e.g., HILIC × RP), can offer a powerful means to enhance the separation of isomeric GSLs that coelute in 1D methods. Similarly, the integration of IMS enables separation based on collision cross-section, providing an additional dimension of structural resolution.

But separation is not enough. Advances in fragmentation technologies offer a deeper solution. Traditional CID and HCD methods are limited in their ability to resolve glycan branching and linkage position. Emerging approaches such as EID and UVPD, which operate through radical-driven mechanisms, can generate rich fragment ion series that include both glycan- and ceramide-specific ions in a single spectrum. These technologies allow for accurate molecular-level annotation rather than approximate class-level guesses and should be prioritized in both instrument development and routine workflows.

#### 6.3.2 Improving Extraction Methods for Highly Glycosylated GSL Species

Alongside instrumental advances, there is a critical need to revisit and improve extraction protocols, particularly for highly glycosylated, polysialylated gangliosides, or sulfated GSLs. These molecules have a large glycan tail relative to the lipid part, e.g., as shown in the example of making them less amenable to extraction by traditional biphasic solvent systems (e.g., Folch or Bligh–Dyer). These methods tend to preferentially recover neutral or weakly polar lipids, while highly glycosylated GSLs may partition poorly, degrade, or become trapped at the interface. More recent monophasic extraction protocols (e.g., methanol–water or MTBE-based systems) and solid-phase extraction techniques using polar stationary phases have shown promise for retaining labile or hydrophilic species. Nevertheless, systematic evaluation and standardization of these approaches in the context of GSL lipidomics remain lacking. Future protocols should aim to maximize recovery across the GSL spectrum, including those with large or acidic headgroups, without introducing bias toward any specific subclass.

#### 6.3.3 Expanding Rule-Based Annotation Tools

Another urgent challenge is the heavy reliance on spectral library matching for GSL identification. While spectral libraries like LipidBlast, GNPS, and HMDB are helpful and have recently even included CCS values for GSL,^159,178^ they remain incomplete, poorly validated for GSLs mainly due to the lack of commercially available standards, and also instrument-specific. Moreover, library matching often promotes overannotation, assigning structures based on similarity rather than evidence. Instead, the field must move toward integrating rule-based annotation frameworks in which diagnostic ions and fragmentation rules drive structural assignments.

Tools such as LDA, LipidMatch, and DANGO offer promising models, applying fragmentation rules to determine the glycan composition, ceramide structure, and even hydroxylation states. However, broader adoption and further development are needed. These tools must support custom rule sets, integrate with diverse instrument platforms, and output confidence-level metrics to make annotation uncertainty explicit and traceable.

Moreover, annotation pipelines must support, or at least introduce an option for, the sum composition nomenclature discussed earlier rather than forcing shorthand class assignments like GD1a, which are not justified in most workflows. In addition, the field needs open-source frameworks that enable researchers to contribute new models, validate them across platforms, and track annotation evolution over time.

#### 6.3.4 Enhancing File Compatibility and Software Interoperability

A persistent obstacle to method development in GSL research is the lack of compatibility between file formats and software tools. Proprietary data formats, inconsistent metadata standards, and limited cross-platform functionality prevent researchers from fully utilizing the available computational tools. To address this, efforts should focus on adopting open data standards (e.g., mzML and mzTab-M), improving interoperability between tools (e.g., linking mzmine with LipidMatch or LDA), and building modular pipelines that allow users to swap components (e.g., normalization, transformation, and imputation) without reprocessing raw data.

#### 6.3.5 Incorporating Multiomics Validation and Pathway Context

GSLs do not exist in isolation. Their biosynthesis is regulated by a series of glycosyltransferases, ceramide synthases, and lipid transporters, many of which are subject to transcriptional or post-transcriptional regulation. To contextualize GSL changes, lipidomics data should increasingly be integrated with transcriptomics, proteomics, and glycomics. This can help validate whether observed GSL shifts reflect actual metabolic reprogramming or are artifacts of sample preparation.

For instance, if an increase in the LacCer species is observed, one should confirm whether the relevant β-1,4-galactosyltransferase (B4GALT5/6) is upregulated. Similarly, if ganglioside diversity expands, transcriptomic data can be investigated and validated via an increase in the expression levels of ST3GAL or ST8SIA.^27,179,180^ These cross-validations transform lipidomic signals from mere lists of masses to biologically coherent mechanisms.

Moreover, systems-level pathway tools like Reactome,^181^ the KEGG database,^182^ and LIPID MAPS pathways should be leveraged to map GSL dynamics onto known biosynthetic routes, helping prioritize candidates for follow-up validation and biomarker discovery.

#### 6.3.6 Advancing toward Therapeutic and Clinical Relevance

Finally, as annotation, normalization, and validation improve, so does the clinical potential of GSLs. Numerous GSL subclasses, including GM3, GD2, and LacCer, have been implicated in cancer progression, immune regulation, and neurodegenerative disease. Yet, without rigorous workflows, these biomarkers remain anecdotal or irreproducible.

The standardization of GSL analysis, including preprocessing protocols, sum composition nomenclature, rule-based annotation, and orthogonal validation, will be crucial for the transition of GSLs from exploratory markers to diagnostic tools or therapeutic targets. Emerging platforms such as GSL-based vaccines, monoclonal antibodies, and ceramide synthase modulators stand to benefit immensely from this standardization.

Moreover, collaborative networks, including multilab consortia, open data platforms, and multiomics integration efforts, will be critical in translating GSL lipidomics into actionable insights in cancer, metabolic disease, infection, and beyond.

In conclusion, the next phase of GSL research must be both critical and constructive. We must question the assumptions underlying current annotation and normalization practices, adopt new technologies, embrace more biologically aligned workflows, and think beyond the mass spectrum toward mechanisms, interactions, and ultimately clinical translation. Figure 8 highlights that confident and biologically relevant GSL annotation requires the convergence of multiple, complementary analytical techniques, including chromatographic orthogonality, polarity-dependent MS acquisition, diverse fragmentation strategies, and postannotation validation steps.

**Figure 8:**
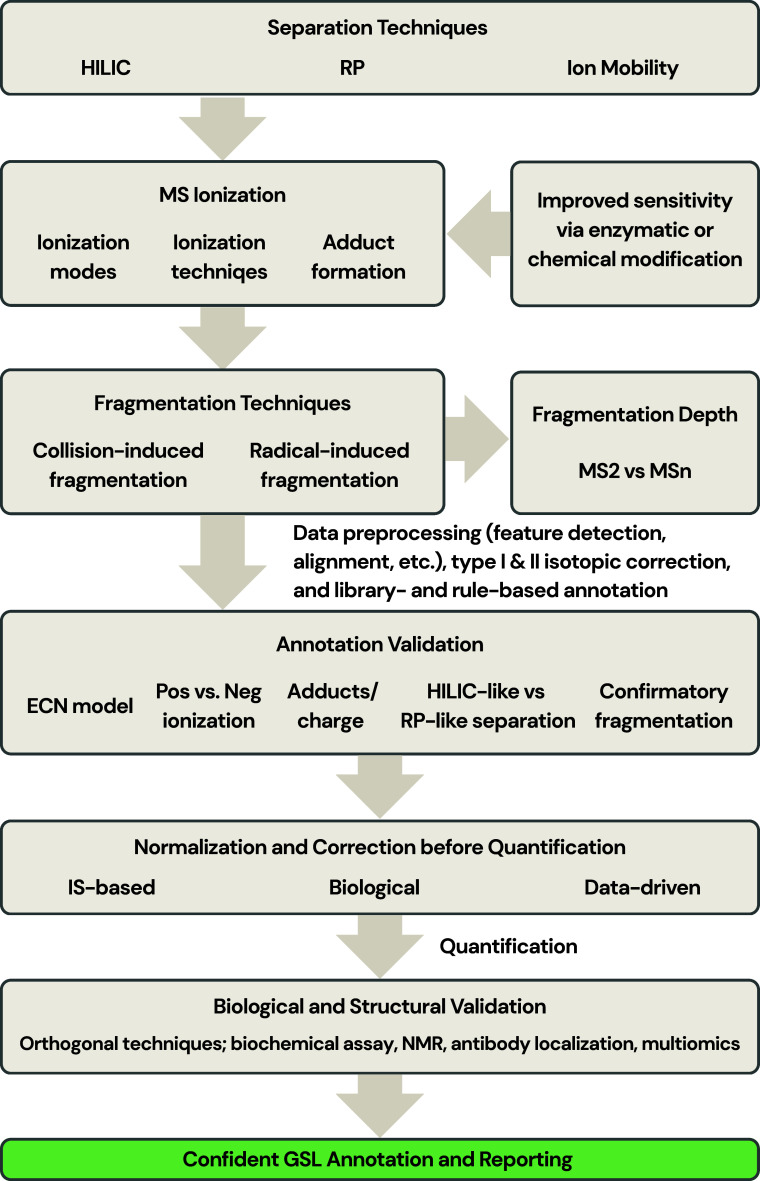
Overview of the multidimensional analytical workflow required for confident glycosphingolipid annotation.
